# ELL targets c-Myc for proteasomal degradation and suppresses tumour growth

**DOI:** 10.1038/ncomms11057

**Published:** 2016-03-24

**Authors:** Yu Chen, Chi Zhou, Wei Ji, Zhichao Mei, Bo Hu, Wei Zhang, Dawei Zhang, Jing Wang, Xing Liu, Gang Ouyang, Jiangang Zhou, Wuhan Xiao

**Affiliations:** 1 The Key Laboratory of Aquatic Biodiversity and Conservation, Institute of Hydrobiology, Chinese Academy of Sciences, 430072 Wuhan, China; 2 State Key Laboratory of Freshwater Ecology and Biotechnology, Institute of Hydrobiology, Chinese Academy of Sciences, 430072 Wuhan, China

## Abstract

Increasing evidence supports that ELL (eleven–nineteen lysine-rich leukaemia) is a key regulator of transcriptional elongation, but the physiological function of *Ell* in mammals remains elusive. Here we show that ELL functions as an E3 ubiquitin ligase and targets c-Myc for proteasomal degradation. In addition, we identify that UbcH8 serves as a ubiquitin-conjugating enzyme in this pathway. Cysteine 595 of ELL is an active site of the enzyme; its mutation to alanine (C595A) renders the protein unable to promote the ubiquitination and degradation of c-Myc. ELL-mediated c-Myc degradation inhibits c-Myc-dependent transcriptional activity and cell proliferation, and also suppresses c-Myc-dependent xenograft tumour growth. In contrast, the ELL(C595A) mutant not only loses the ability to inhibit cell proliferation and xenograft tumour growth, but also promotes tumour metastasis. Thus, our work reveals a previously unrecognized function for ELL as an E3 ubiquitin ligase for c-Myc and a potential tumour suppressor.

## Abstract (web summary)

The expression of the oncogene Myc is carefully controlled and dysregulation often leads to cancer. Here, the authors describe an E3 ligase for Myc—ELL—and show that it likely controls the ubiquitination and degradation of Myc.

## Graphical abstract

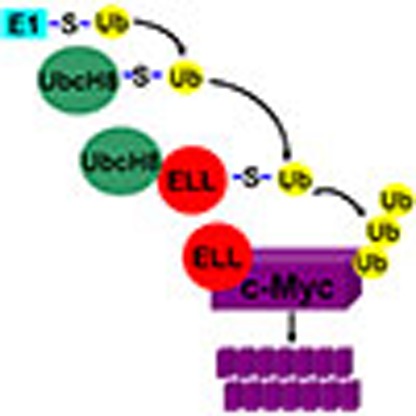

The product of the *eleven–nineteen lysine-rich leukaemia* (*ELL*) gene was first identified as a translocation partner of the mixed-lineage leukaemia (*MLL*) gene in acute myeloid leukaemia (AML)^1^. Subsequent studies identified *ELL* as a transcription elongation factor that could increase the rate of transcriptional elongation by RNA polymerase II *in vitro*, and later *in vivo* studies revealed its association with transcriptionally active loci^2^,^3^. ELL is a part of two distinct elongation complexes, the super elongation complex (SEC) and the little elongation complex (LEC)^4^,^5^,^6^. The SEC plays several important functions, such as *HSP70* induction^7^,^8^, *HOX* gene dysregulation^7^ and HIV transcription activation^9^,^10^. The LEC functions at the initiation and elongation phases of *snRNA* gene transcription^5^,^11^.

In mammals, *ELL* is required for early embryogenesis^12^. Moreover, ELL has been identified as a partner of steroid receptors, hypoxia-inducible factor 1-alpha (HIF-1α), E2F1 and the TFIIH complex, modulating their binding partner's activity^13^,^14^,^15^,^16^.

The proto-oncogene, *c-Myc*, is frequently translocated in multiple myeloma and is highly amplified or mutated in many different human cancers^17^. The *c-Myc* gene encodes a multifunctional transcription factor that plays important roles in regulating the expression of genes contributed to tumorigenesis, tumour maintenance as well as tumour metastasis^17^. One of the most prominent mechanisms to degrade c-Myc is through the ubiquitin-proteasome pathway^18^,^19^. Fbw7 is the best studied E3 ubiquitin ligase for mediating c-Myc inhibition through degradation^20^,^21^. Another RING-FINGER E3 ligase, Skp2, recognizes a conserved sequence element in the amino terminus of c-Myc (MBII) and HLH-LZ motifs (amino acids 367–439), promoting its poly-ubiquitination and degradation^22^,^23^. The third RING-FINGER E3 ligase, β-TrCP, binds to the amino terminus of c-Myc and uses the UbcH5 ubiquitin-conjugating enzyme (E2) to form heterotypic polyubiquitin chains on c-Myc^24^. The only homologous to E6-AP C-terminus (HECT) E3 ligase reported for c-Myc, HectH9, ubiquitinates c-Myc, forming a lysine 63-linked polyubiquitin chain^25^, which does not trigger c-Myc degradation but, instead, is required for the transactivation of multiple target genes by c-Myc^25^.

As one of the classic oncogenes, *c-Myc* is overexpressed in about 70% of human tumours; however, only 20% of these tumours exhibit *c-Myc* gene amplification or translocation^18^. Thus, the deregulation of E3 ubiquitin ligase may contribute to the overexpression of c-Myc observed in human tumours. In fact, aberrant expression and/or mutation of some E3 ligases of c-Myc have been reported in tumours^18^,^26^,^27^,^28^.

In this study, we reveal a previously unrecognized function for ELL as an E3 ubiquitin ligase for c-Myc.

## Results

### ELL promotes c-Myc degradation

**Figure 1:**
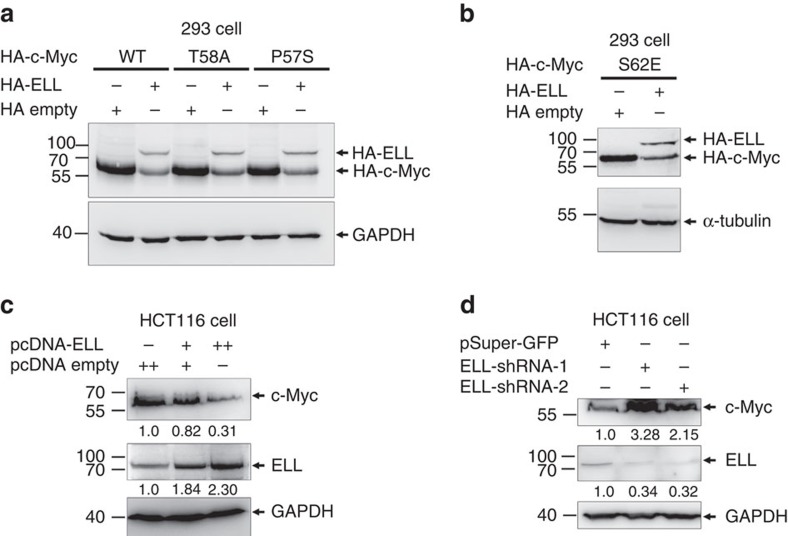
ELL induces c-Myc degradation. (**a**) Co-transfection of ELL induces the protein degradation of wild-type c-Myc as well as the T58A and P57S mutants in HEK293 cells. (**b**) Co-transfection of ELL induces the protein degradation of the c-Myc S62E mutant. (**c**) Overexpression of ELL in HCT116 cells reduces endogenous c-Myc protein degradation in a dose-dependent manner. (**d**) shRNA-mediated ELL knockdown in HCT116 cells by ELL-shRNAs (ELL-shRNA-1 and ELL-shRNA-2) enhances endogenous c-Myc protein level.

Using an anti-Myc antibody (9E10, Santa Cruz) to detect Myc-tagged proteins in transfected cells, we noticed that it could also detect a band of ∼67 kDa, which was likely endogenous c-Myc. Intriguingly, the endogenous c-Myc band disappeared with Myc-ELL overexpression. This phenomenon led us to hypothesize that ELL might mediate c-Myc degradation. Ectopic expression of HA-ELL reduced HA-c-Myc protein levels (Fig. 1a). Because phosphorylation at Ser 62 stabilizes c-Myc, whereas subsequent phosphorylation at Thr 58 is required for c-Myc degradation^29^, we next examined whether ELL promoted degradation of a c-Myc Thr 58 phosphorylation-dead mutant, T58A, a Ser 62 constitutive-phosphorylation mutant, S62E, as well as a Burkitt's lymphoma-derived Myc mutant, P57S. Overexpression of ELL induced degradation of all the mutants as well as the wild-type c-Myc (Fig. 1a,b). These results suggest that c-Myc phosphorylation is dispensable for ELL-mediated degradation.

To further examine the effects of ELL on the stability of the wild-type c-Myc, as well as that of the c-Myc mutants, we co-transfected the wild-type or mutant c-Myc with HA-ELL or HA empty vector control in the presence of cycloheximide (50 μg ml^−1^) and performed time-cause assays. Overexpression of ELL accelerated degradation of the wild-type and mutant c-Myc (Supplementary Fig. 1A–E). Furthermore, overexpression of ELL in HCT116 cells induced endogenous c-Myc degradation in a dose-dependent manner (Fig. 1c). In contrast, knockdown of ELL in HCT116 cells enhanced endogenous c-Myc stability (Fig. 1d) even in the presence of 50 μg ml^−1^ cycloheximide (Supplementary Fig. 1F).

To determine whether ELL can affect the transcription of *c-Myc*, we overexpressed or knocked down of ELL in HCT116 cells and examined the mRNA levels of *c-Myc*. Neither overexpression of ELL nor knockdown of ELL had obvious effect on c-Myc mRNA levels (Supplementary Fig. 2A and B).

Taken together, these results suggest that ELL induces c-Myc protein degradation in a manner that is not dependent on c-Myc phosphorylation and new protein synthesis.

### ELL interacts with c-Myc *in vivo* and *in vitro*

**Figure 2:**
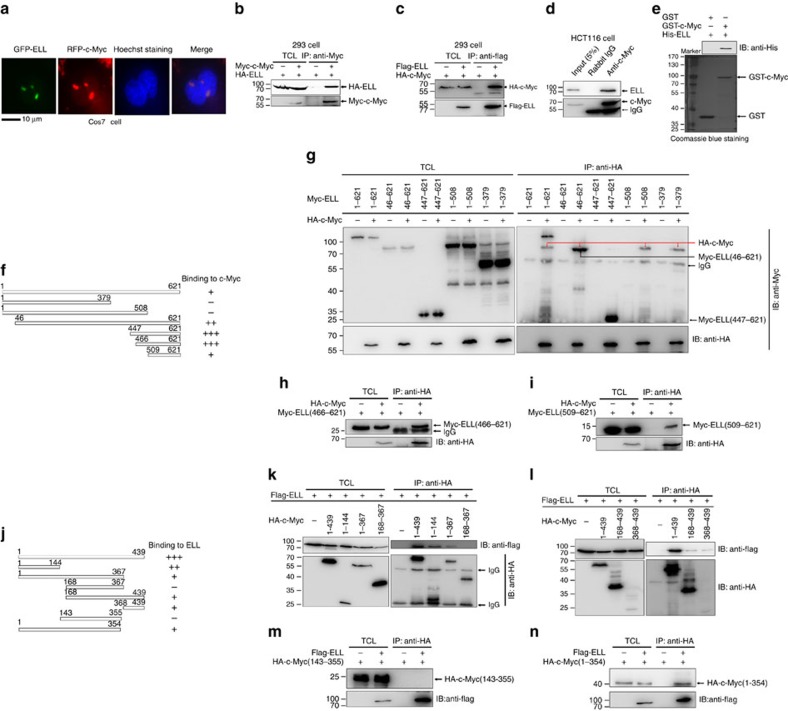
ELL interacts with c-Myc. (**a**) RFP-tagged c-Myc co-localizes with GFP-tagged ELL in the nucleus after co-transfection into Cos7 cells. The nucleus were stained by Hoechst 33342. (**b**) Co-immunoprecipitation of human c-Myc with human HA-ELL in HEK293T cells transfected with the indicated plasmids. (**c**) Co-immunoprecipitation of human Flag-ELL with human HA-c-Myc in HEK293T cells transfected with the indicated plasmids. (**d**) Co-immunoprecipitation of endogenous ELL with endogenous c-Myc in HCT116 cells. (**e**) ELL directly interacts with c-Myc. (**f**) Schematic of the ELL domains. The extent of the interaction between c-Myc and the ELL domains is indicated by the number of plus signs. (**g**–**i**) Co-immunoprecipitation of human HA-c-Myc with Myc-tagged ELL domains in HEK293T cells transfected with the indicated plasmids. (**j**) Schematic of c-Myc domains. The extent of the interaction between ELL and the c-Myc domains is indicated by the number of plus signs. (**k**–**n**) Co-immunoprecipitation of human Flag-ELL and HA-tagged c-Myc domain in HEK293T cells transfected with the indicated plasmids. IB, immunoblotting; TCL, total cellular lysates.

To gain insight into the mechanisms by which ELL induces c-Myc degradation, we examined whether ELL interacts with c-Myc. RFP-tagged c-Myc co-localized with GFP-tagged ELL in the nucleus of Cos7, HEK293T and HCT116 cells, forming nuclear speckles (Fig. 2a; Supplementary Fig. 3A). To examine whether RFP-c-Myc co-localized with GFP-ELL in the nucleolus, we co-transfected RFP-c-Myc with GFP-tagged BM5, a nucleolus marker^30^, into Cos7 cells with or without ectopic expression of ELL. Notably, the speckles formed by GFP-BM5 were clearly separated from the speckles formed by co-localization of RFP-c-Myc and HA-ELL (Supplementary Fig. 3B), thus ruling out the possibility that ELL co-localized with c-Myc in the nucleolus. Next, we examined whether ELL co-localize with Max or Mxd. In the presence of HA-c-Myc, GFP-ELL co-localized with RFP-Max (Supplementary Fig. 3C). However, in the absence or presence of c-Myc, GFP-ELL did not co-localize with RFP-Mxd (Supplementary Fig. 3D).

Co-immunoprecipitation assays showed that Myc-tagged c-Myc could pull down HA-ELL after co-transfection into HEK293 cells (Fig. 2b). Similarly, Flag-ELL could also pull down HA-c-Myc (Fig. 2c). Co-immunoprecipitation assays in HCT116 cells using a polyclonal anti-c-Myc antibody (A0309, ABclonal) indicated that c-Myc interacted with endogenous ELL (Fig. 2d). Moreover, glutathione S-transferase (GST)-pull-down assays using GST-tagged c-Myc and His-tagged ELL expressed in *Escherichia coli* (*E. coli*) showed that GST-tagged c-Myc could pull down His-tagged ELL (Fig. 2e). These results suggest that endogenous ELL directly interacts with c-Myc.

We subsequently mapped the domains of ELL and c-Myc that are responsible for their interaction (Fig. 2f,j). The C terminus of ELL (amino acids (aa) 466–621) was crucial for interaction with c-Myc (Fig. 2g–i), and the N terminus (aa 1–144) and C terminus (aa 368–439) of c-Myc were required for interaction with ELL (Fig. 2k–n). Notably, the DNA-binding domain of c-Myc (aa 143–355) did not interact with ELL (Fig. 2m), but the mutant with Max-binding domain (aa 1–354) deletion could still bind to ELL.

### ELL is an E3 ubiquitin ligase

**Figure 3:**
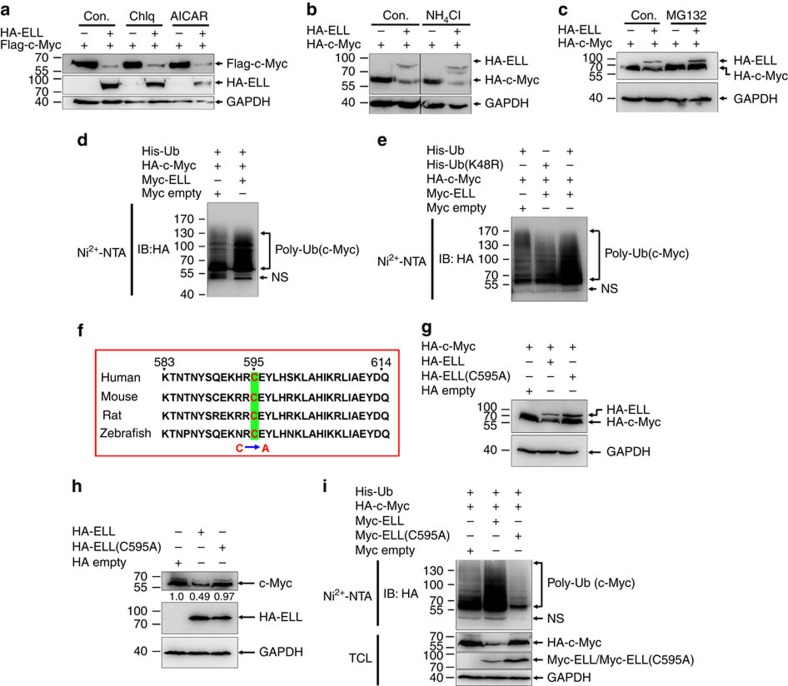
ELL mediates c-Myc proteasomal degradation. (**a**) The lysosomal inhibitor, chloroquine diphosphate (Chlq, 10 μM), and the autophagy inhibitor, AICAR (0.2 mM), do not block ELL-induced Flag-c-Myc degradation. (**b**) The lysosomal inhibitor, NH_4_Cl (25 mM), does not block ELL-induced HA-c-Myc degradation. (**c**) ELL-induced degradation of HA-c-Myc was blocked by the proteasome inhibitor, MG132 (20 μM). (**d**) ELL enhances the poly-ubiquitination of c-Myc. (**e**) ELL does not enhance the poly-ubiquitination of c-Myc when ubiquitin K48 is mutated to R (K48R). (**f**) Alignment of partial ELL sequences (583–614 amino acids in human ELL) from human, mouse, rat and zebrafish. The cysteine at position 595 (C595) of human ELL was mutated to alanine (C595A). (**g**) The ELL(C595A) mutant does not induce c-Myc degradation. (**h**) Overexpression of the ELL(C595A) mutant does not obviously alter endogenous c-Myc protein level. (**i**) The ELL(C595A) mutant does not enhance the poly-ubiquitination of c-Myc. NS, non-specific band.

To characterize the type of protein degradation mediated by ELL, we took advantage of inhibitors, including chloroquine (lysosomal proteolysis inhibitor), NH_4_Cl (lysosomal proteolysis inhibitor), AICAR (macro-autophagy inhibitor) and MG132 (proteasome inhibitor). Only the proteasome inhibitor, MG132, could block ELL-mediated c-Myc degradation (Fig. 3a–c), suggesting that ELL promotes c-Myc degradation via the proteasome pathway. To validate that ELL indeed participates in the proteasomal degradation of c-Myc, we performed *in vivo* ubiquitination assays by co-transfecting His-ubiquitin and HA-c-Myc into HEK293T cells together with a Myc empty vector or Myc-ELL. Overexpression of ELL enhanced the poly-ubiquitination of c-Myc (Fig. 3d). Given the role of lysine (K) 48-linked poly-ubiquitination in proteolysis^19^, we performed an ubiquitination assay using an ubiquitin mutant, Ub(K48R), which cannot form K48-conjugated polyubiquitin chains. ELL strongly induced the poly-ubiquitination of c-Myc in the presence of wild-type ubiquitin, but not K48R ubiquitin (Fig. 3e), suggesting that ELL promotes the formation of K48-linked polyubiquitin chains on c-Myc.

These data led us to further hypothesize that ELL might have E3 ubiquitin ligase activity. We performed domain mapping of ELL and analysed c-Myc degradation, we found that aa 583–614 of ELL is required for ELL-mediated c-Myc degradation (Fig. 2g; Supplementary Fig. 4). Within this region, there is only one cysteine (C) located at position 595 (C595), which is evolutionarily conserved from zebrafish to human (Fig. 3f). Because the HECT and RBR-domain E3 ligases have a cysteine active site required for their catalytic activity^19^, we examined whether ELL still functions as an E3 ligase if C595 of ELL is mutated to alanine (C595A). The ELL(C595A) mutant did not promote c-Myc degradation even though it could still interact with c-Myc (Fig. 3g; Supplementary Fig. 5). Moreover, compared with wild-type ELL, overexpression of the ELL(C595A) mutant had no obvious effect on the stability of endogenous c-Myc (Fig. 3h). In contrast to that of wild-type ELL, overexpression of the ELL(C595A) mutant diminished the poly-ubiquitination of c-Myc (Fig. 3i). These results suggest that ELL might have E3 ubiquitin ligase activity and that cysteine 595 is an active site.

**Figure 4:**
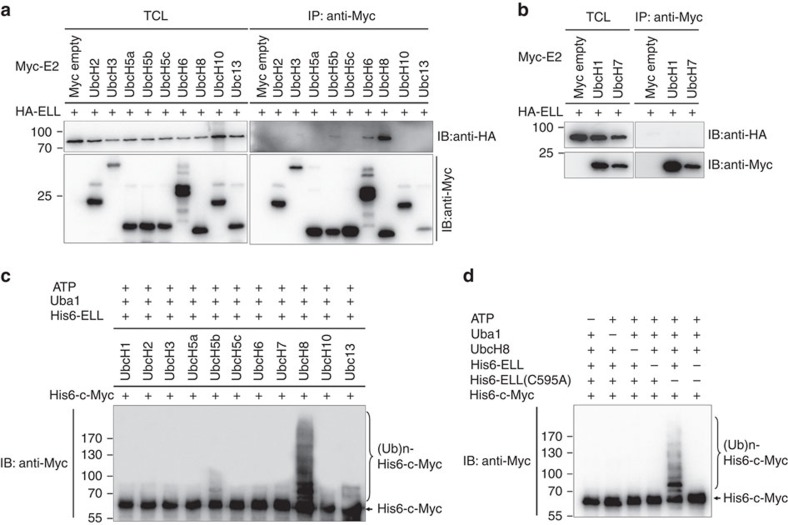
ELL function as an E3 ubiquitin ligase. (**a**,**b**) Co-immunoprecipitation assays between ELL and 11 E2 ubiquitin-conjugating enzymes show that UbcH8 has the strongest binding ability to ELL, UbcH6 and UbcH5b have weaker binding ability to ELL; other E2 enzymes do not interact with ELL. (**c**) *In vitro* ubiquitination assays show that in the presence of UbcH8 (E2), the purified ELL expressed in *E. coli* induces poly-ubiquitination of purified c-Myc expressed in *E. coli* most efficiently; and in the presence of UbcH5b (E2), ELL induces poly-ubiquitination of c-Myc mildly. (**d**) *In vitro* ubiquitination assays show that the purified ELL expressed in *E. coli* induces poly-ubiquitination of purified c-Myc expressed in *E. coli* even in the presence of UbcH8 (E2) and ATP, but ELL(C595A) does not. Ubc1 (E1) and biotinylated ubiquitin were added to all of the reactions. ub, ubiquitin.

To evaluate whether ELL is a *bona fide* E3 ubiquitin ligase, we conducted *in vitro* ubiquitination assays using an ubiquitinylation kit (UW9920, BioMol). To define which E2 enzymes are involved in ELL mediating c-Myc proteasomal degradation, we first cloned 11 E2 enzymes indicated in the kit into CMV-Myc expression vector and examined their binding ability to ELL. Three E2 enzymes including UbcH8, UbcH6 and UbcH5b could bind to ELL, and UbcH8 has the strongest binding ability, UbcH5b has the weakest binding ability, but other eight E3 enzymes do not bind to ELL at all (Fig. 4a,b). Subsequently, we expressed His6-ELL, His6-ELL(C595A) and His6-c-Myc in *E. coli* and purified them by affinity purification (Supplementary Fig. 6A and B). Then, we performed ubiquitination assays according to the protocol provided by the kit (UW9920, BioMol) with some modifications. Only adding of UbcH8 in the reaction caused dramatic poly-ubiquitination of His6-c-Myc in the presence of His6-ELL, and adding of UbcH5b caused poly-ubiquitination of His6-c-Myc at very low level, but adding of other E2 enzymes did not cause obvious poly-ubiquitination of His6-c-Myc (Fig. 4c). Moreover, compared with adding His6-ELL to the reaction, adding of His6-ELL(C595A) did not induce poly-ubiquitination of His6-c-Myc in the presence of UbcH8 (Fig. 4d). These data not only suggest that ELL itself has E3 ligase activity, but also indicate that UbcH8 serves as an E2 ubiquitin-conjugating enzyme in the pathway. In addition, the ELL(C595A) mutant loses E3 ubiquitin ligase activity.

**Figure 5:**
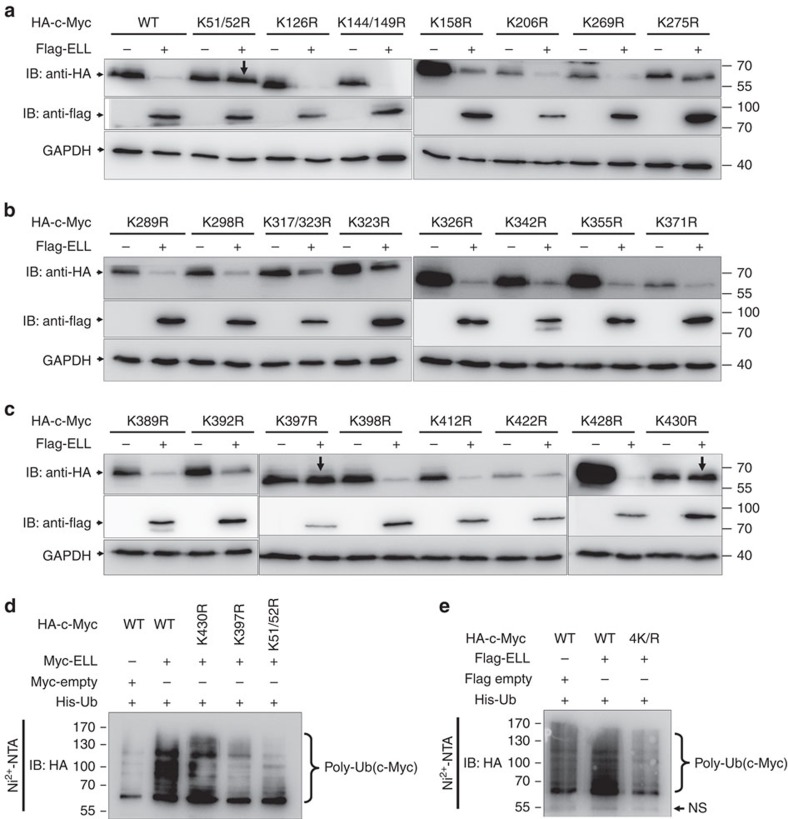
ELL targets c-Myc at K51/K52/K397/K430. (**a**–**c**) The HA-tagged wild-type c-Myc and 23 HA-tagged c-Myc mutants were co-transfected with Flag-ELL into HEK293T cells, the expressions of wild-type c-Myc and c-Myc mutants were detected by anti-HA antibody. ELL does not induce degradation of the K51/52R, K397R and K430R c-Myc mutants. (**d**) Compared with that of the wild-type c-Myc, the poly-ubiquitination of the K51/52R, K397R and K430R mutants by ELL was diminished. (**e**) ELL does not promote poly-ubiquitination of the c-Myc(4K/R) mutant.

As a typical E3 ubiquitin ligase usually catalyses its targets at lysine (K) residue(s) to form polyubiquitin chains^19^, we next determined which lysine residues in c-Myc are catalysed by ELL. Given that ELL promotes wild-type c-Myc degradation, we used the protein degradation efficiency by ELL to monitor the potential ubiquitination site(s) in c-Myc. Overexpression of ELL did not promote degradation of the K51/52R, K397R and K430R mutants (Fig. 5a–c; Supplementary Fig. 7A and B). Compared with that of the wild-type c-Myc, ELL-induced poly-ubiquitination of the K51/52R, K397R and K430R mutants, as well as that of the four-site mutant 4K/R (K51/52/397/430R), was reduced (Fig. 5d,e). However, all the mutants could still interact with ELL (Supplementary Fig. 7C). These data suggest the K51/52, K397 and K430 in c-Myc are key sites for c-Myc poly-ubiquitination catalysed by ELL. In addition, it appears that simultaneous ubiquitination of these key sites by ELL is required for c-Myc degradation because two single-site mutants (K397R, K430R) and one double-site mutant (K51/52R) were not degraded by ELL efficiently (Fig. 5a–c).

Collectively, these results suggest that ELL is a *bona fide* E3 ubiquitin ligase, targeting c-Myc for proteasomal degradation, and that UbcH8 serves as an E2 ubiquitin-conjugated enzyme in this pathway. In addition, cysteine 595 in ELL serves as an active site.

### ELL inhibits c-Myc transcriptional activity

**Figure 6:**
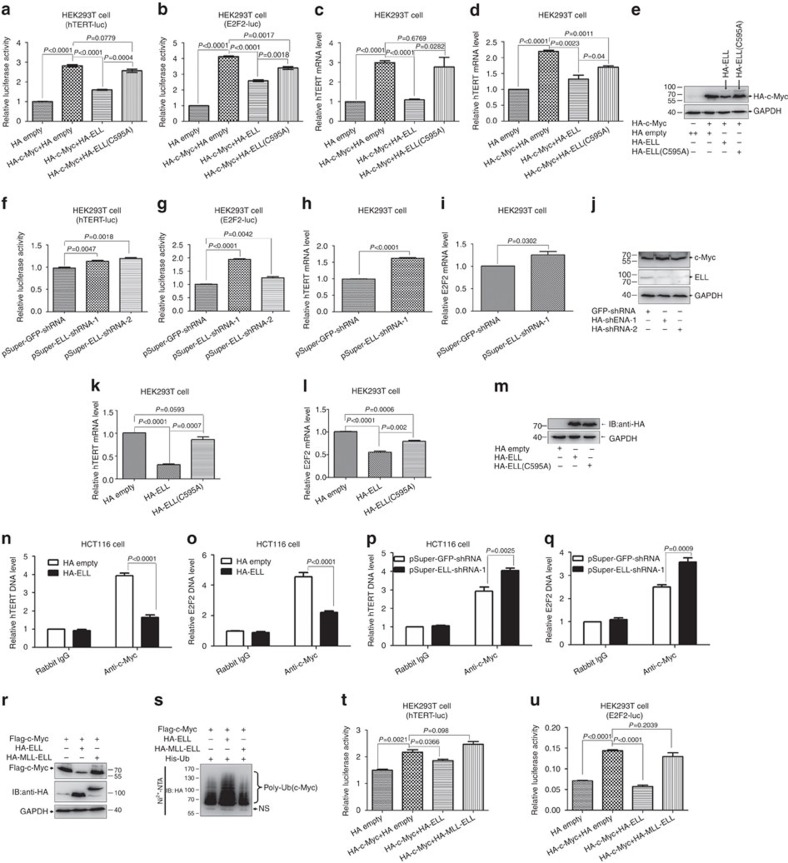
ELL inhibits c-Myc transcriptional activity. (**a**) Overexpression of ELL suppresses *hTERT* promoter reporter activity induced by c-Myc (*P*<0.0001, *t*-test), but overexpression of ELL(C595A) does not (*P*=0.0779, *t*-test). (**b**) Overexpression of ELL and ELL(C595A) suppresses *E2F2* promoter reporter activity induced by c-Myc (*P*<0.0001 and *P*=0.0017, respectively, *t*-test). (**c**) Overexpression of ELL inhibits the expression of *hTERT* activated by c-Myc (*P*<0.0001, *t*-test), but overexpression of ELL(C595A) does not (*P*=0.6769, *t*-test). (**d**) Overexpression of ELL and ELL(C595A) inhibits the expression of *E2F2* activated by c-Myc (*P*=0.0023 and *P*=0.0011, respectively, *t*-test). (**e**) The expression of HA-ELL, HA-ELL(C595A) and HA-c-Myc in HEK293T cells is confirmed. (**f**,**g**) Knockdown of *ELL* in HEK293T cells by pSuper-ELL-shRNA1 and pSuper-ELL-shRNA2 enhances *hTERT* (**f**) and *E2F2* (**g**) promoter reporter activity. (**h**,**i**) Knockdown of *ELL* in HEK293T cells by pSuper-ELL-shRNA1 increases *hTERT* (**h**) and *E2F2* (**i**) mRNA levels. (**j**) pSuper-ELL-shRNA1- and pSuper-ELL-shRNA2-mediated knockdown of ELL is confirmed. (**k**) Overexpression of ELL inhibits *hTERT* expression (*P*<0.0001, *t*-test), but overexpression of ELL(C595A) does not (*P*=0.0593, *t*-test). (**l**) Overexpression of ELL and ELL(C595A) inhibits *E2F2* expression(*P*<0.0001 and *P*=0.0006, respectively, *t*-test). (**m**) The expression of HA-ELL and HA-ELL(C595A) in HEK293T cells is confirmed by western blot analysis. (**n**) Overexpression of ELL in HCT116 cells reduces c-Myc binding to h*TERT* promoter (*P*<0.0001, *t*-test). (**o**) Overexpression of ELL in HCT116 cells reduces c-Myc binding to *E2F2* promoter (*P*<0.0001, *t*-test). (**p**) Knockdown of ELL in HCT116 cells by pSuper-ELL-shRNA-1 enhances c-Myc binding to h*TERT* promoter (*P*=0.0025, *t*-test). (**q**) Knockdown of ELL in HCT116 cells by pSuper-ELL-shRNA-1 enhances c-Myc binding to *E2F2* promoter (*P*=0.0025, *t*-test). (**r**) Overexpression of HA-MLL-ELL does not induce Flag-c-Myc degradation. (**s**) Overexpression of HA-MLL-ELL does not promote c-Myc poly-ubiquitination. (**t**,**u**) Overexpression of HA-MLL-ELL has no inhibitory effect on *hTERT* (**t**) or *E2F2* (**u**) promoter reporter activity induced by c-Myc. Data are presented as mean ±s.e.m. of three independent experiments performed in triplicate.

To evaluate the biological consequences of ELL-mediated c-Myc degradation, we examined the effect of ELL on c-Myc-dependent transactivation. Using a mammalian one-hybridization system by co-transfecting HA-ELL together with c-Myc fused to the GAL4 DNA-binding domain, overexpression of ELL significantly inhibited the transcriptional activity of c-Myc (Supplementary Fig. 8A). Next, we examined the effect of ELL on the transactivation of two well-defined c-Myc target genes, *hTERT*^31^ and *E2F2* (ref. 32). c-Myc activated the *hTERT* promoter by ∼2.8-fold. But, co-expression of ELL together with c-Myc decreased *hTERT* promoter activity (Fig. 6a). In contrast, co-expression of the ELL(C595A) mutant together with c-Myc had no obvious effect on c-Myc transcriptional activity (Fig. 6a). Similar results were obtained for *E2F2* promoter activity (Fig. 6b) with the exception that co-expression of ELL(C595A) with *c-Myc* suppressed *E2F2* promoter activity (Fig. 6b).

Subsequently, we used semi-quantitative reverse transcription (RT)-PCR to examine the effect of ELL on c-Myc-dependent transactivation. *c-Myc* induced *hTERT* mRNA levels ∼2.8-fold (Fig. 6c), which was reduced with co-expression of *ELL*, but not ELL(C595A) (Fig. 6c). Similar results were obtained for ELL on *E2F2* mRNA level (Fig. 6d). In agreement with the promoter assays, co-expression of the ELL(C595A) mutant with c-Myc suppressed *E2F2* expression (Fig. 6d). The expression of transfected HA-c-Myc, HA-ELL and HA-ELL(C595A) was confirmed (Fig. 6e).

To further explore the influence of ELL on c-Myc-dependent transactivation, we analysed the effect of ELL knockdown by transfection with two ELL-shRNA constructs, pSuper-ELL-shRNA-1 and pSuper-ELL-shRNA-2 (ref. 15). Knockdown of ELL by two ELL-shRNA constructs in HEK293T cells caused an increase in *hTERT* and *E2F2* promoter activity (Fig. 6f,g), and *hTERT* and *E2F2* mRNA levels (Fig. 6h,i). Knockdown of ELL by ELL-shRNAs was confirmed (Fig. 6j).

To determine the effect of overexpression of ELL on endogenous c-Myc activity, we examined expressions of *hTERT* and *E2F2* with overexpression of ELL or ELL(C595A) via transient transfection. Overexpression of ELL reduced *hTERT* mRNA level, but overexpression of ELL(C595A) did not (Fig. 6k). Overexpression of both ELL and ELL(C595A) reduced *E2F2* mRNA level (Fig. 6l). Overexpression of ELL and ELL(C595A) was confirmed (Fig. 6m).

To determine whether transcription regulation by c-Myc is affected by ELL, we performed chromatin immunoprecipitation assays (ChIP). Overexpression of *ELL* reduced c-Myc binding to the promoters of *hTERT* and *E2F2* dramatically (Fig. 6n,o). However, knockdown of ELL by pSuper-ELL-shRNA-1 enhanced c-Myc binding to the promoters of *hTERT* and *E2F2* (Fig. 6p,q).

To determine the physiological relevance of ELL-mediated c-Myc degradation, we examined the effect of the MLL-ELL fusion protein, an oncogenic protein identified in AML^1^, on c-Myc function. Co-expression of MLL-ELL together with c-Myc in HEK293T cells had no obvious effect on c-Myc stability as compared with co-expression with ELL (Fig. 6r). In addition, co-expression of MLL-ELL did not enhance the poly-ubiquitination of c-Myc (Fig. 6s) or *hTERT* and *E2F2* promoter activity (Fig. 6t,u).

We next determined whether the ELL-mediated inhibition of *hTERT* and *E2F2* promoter activity and mRNA expression was dependent on c-Myc. We generated three stable HCT116 cell lines with lentiviruses. The first cell line expressed c-Myc-shRNA, which targets the 5′ untranslated region region of *c-Myc* (Supplementary Fig. 8B). The second and third cell lines were established by re-infecting the first cell line with lentiviruses expressing the wild-type c-Myc or the c-Myc(4K/R) mutant (Supplementary Fig. 8C). In cells with stable c-Myc-knock down, overexpression of ELL had no inhibitory effect on *hTERT* and *E2F2* promoter activity (Supplementary Fig. 8D and E) or mRNA levels (Supplementary Fig. 8F and 8G). On c-Myc restoration, the inhibitory effects of ELL on the activation of *hTERT* and *E2F2* promoter was restored (Supplementary Fig. 8H–K). Notably, similar to that exhibited above (Fig. 6b,d,l), overexpression of the ELL(C595A) mutant still suppressed *E2F2* expression in HCT116 cells with c-Myc restoration (Supplementary Fig. 8I and K), further confirming the inhibitory role of ELL(C595A) on *E2F2*. The expression of HA-ELL and HA-ELL(C595A) was confirmed (Supplementary Fig. 8L). Moreover, in HCT116 cells with c-Myc(4K/R) ectopic expression, the inhibitory effects of ELL on expression of *hTERT* and *E2F2* were not detected by both promoter assays and semi-quantitative RT–PCR assays (Supplementary Fig. 8M–P). The expression of HA-ELL and HA-ELL(C595A) was confirmed (Supplementary Fig. 8Q).

Taken together, these results suggest that ELL inhibits c-Myc-dependent transcriptional activity. In addition, although the enzymatic dead mutant, ELL(C595A), is unable to inhibit the expression of one of c-Myc target, *hTERT*, it retains the ability to inhibit the expression of another c-Myc target, *E2F2*, suggesting that ELL rather than acting as an E3 ubiquitin ligase might differentially inhibit c-Myc transcriptional activity.

Of note, ELL(C595A) had no obvious effect on the interaction between c-Myc and Max (Supplementary Fig. 9). In addition, ELL could not affect the suppressive function of c-Myc on *Gadd45α* expression (Supplementary Fig. 10).

### ELL suppresses cell growth and proliferation

ELL has been shown to induce apoptosis^33^. However, we did not observe a relative higher apoptotic ratio in HCT116 cells with lentivirus-mediated ELL overexpression compared with the control cells (Supplementary Fig. 11A and B). So, stable overexpression of ELL might not induce cell apoptosis.

**Figure 7:**
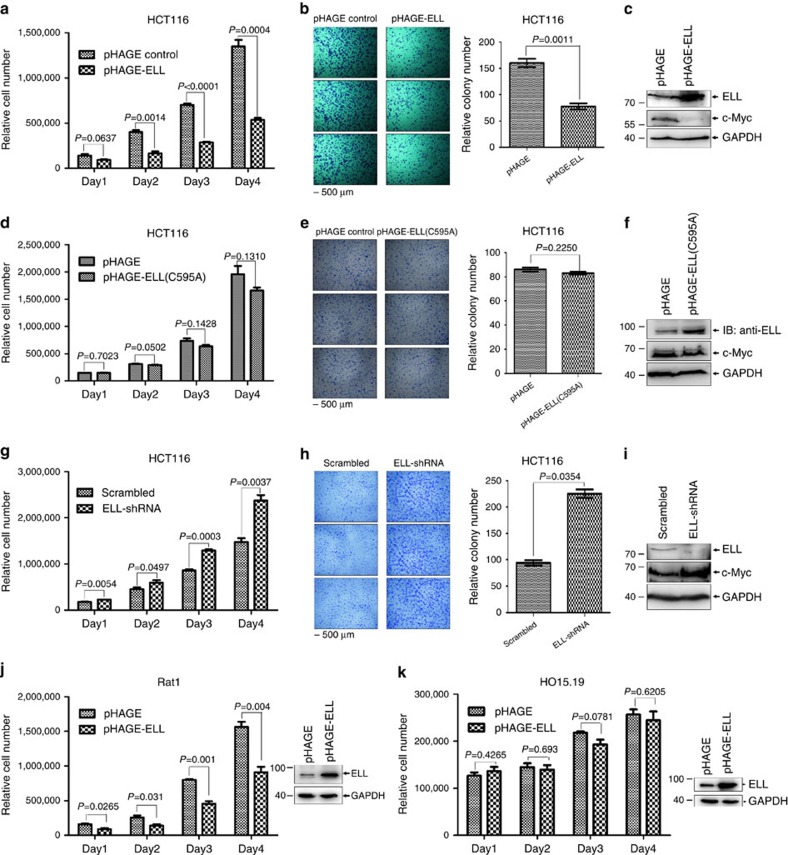
ELL inhibits cell proliferation. (**a**) Overexpression of ELL via lentivirus infection significantly inhibits HCT116 cell proliferation. (**b**) Lentivirus-mediated overexpression of ELL significantly inhibits the colony formation of HCT116 cells (*P*=0.0011, *t*-test). (**c**) Expression of ELL in HCT116 cells is confirmed by western blot analysis. (**d**) Overexpression of the ELL(C595A) mutant via lentivirus infection has no obvious effect on HCT116 cell proliferation. (**e**) Overexpression of the ELL(C595A) mutant via lenti-virus infection has no obvious effect on the colony formation of HCT116 cells (*P*=0.2250, *t*-test). (**f**) Expression of the ELL(C595A) mutant in HCT116 cells is confirmed by western blot analysis. (**g**,**h**) ELL-shRNA-mediated knockdown of ELL enhances HCT116 cell proliferation (**g**) and colony formation (*P*=0.0354, *t*-test) (**h**). (**i**) Knockdown of ELL in HCT116 cells is confirmed by western blot analysis. (**j**) Overexpression of ELL in Rat1 cells (wild-type c-Myc) via lentivirus infection significantly inhibits cell proliferation. (**k**) Overexpression of ELL in HO15.19 cells (c-Myc-null) via lentivirus infection has no obvious effect on cell proliferation. Data are presented as mean ±s.e.m. of three independent experiments performed in triplicate.

To further demonstrate the biological function of ELL in mediating c-Myc degradation and inhibiting its transcriptional activity, we examined its effect on cell proliferation using three stable HCT116 cell lines generated by lentivirus infection, control, ELL and ELL(C595A). Compared with the control cells, HCT116 cells with ELL overexpression proliferated much slower from day 2 (Fig. 7a), which was further validated by colony formation assays (Fig. 7b). The expression of ELL in HCT116 cells was confirmed (Fig. 7c). In contrast, the proliferation rate of the HCT116 cells with stable ELL(C595A) mutant expression is similar to that of control cells (Fig. 7d), which was also confirmed by colony formation assays (Fig. 7e). The expression of ELL(C595A) was confirmed (Fig. 7f). Conversely, the HCT116 cells with stable ELL knockdown proliferated faster as compared with that of control cells expressing scrambled shRNA (Fig. 7g,h). The efficiency of ELL-shRNA-mediated knockdown was confirmed (Fig. 7i).

To further determine whether the effect of ELL on cell proliferation is dependent on *c-Myc*, we took advantage of Rat1 and HO15.19 cells. Rat1 cells contain wild-type *c-Myc*, but HO15.19 cells are derived from Rat1 with targeted disruptions of both c-Myc gene copies^22^,^34^,^35^,^36^,^37^. Overexpression of ELL via lentivirus infection suppressed Rat1 cell proliferation significantly (Fig. 7j). In contrast, overexpression of ELL had no obvious effect on HO15.19 cell proliferation (Fig. 7k).

Taken together, these data suggest that ELL can inhibit cell proliferation, which was mediated by c-Myc.

### ELL suppresses xenograft tumour growth

**Figure 8:**
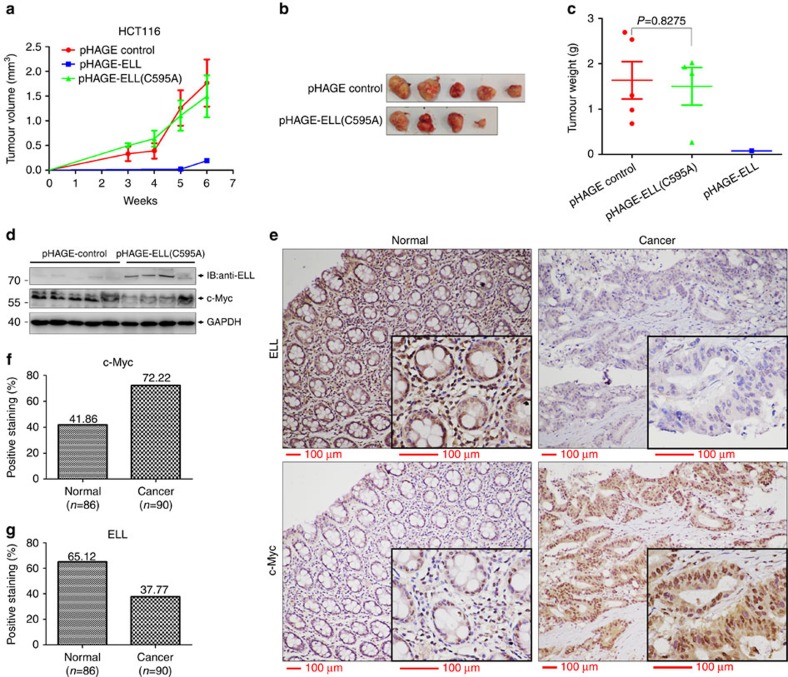
*ELL* suppresses colon tumour xenograft growth. (**a**) Overexpression of ELL inhibits HCT116 tumour xenograft growth in nude mice (*n*=5), but overexpression of the ELL(C595A) mutant has no obvious effect (*n*=5 or *n*=4, after week 4). Data are presented as mean ±s.e.m. (**b**) Tumours harvested from the nude mice after 6 weeks. (**c**) The weight of the tumours after 6 weeks. control, *n*=5; ELL(C595A), *n*=4 (one mouse died at week 4); ELL, *n*=1 (no tumour grew in other four mice). Data are presented as mean ±s.e.m. (**d**) Expression of ELL, ELL(C595A) and c-Myc in the tumours is confirmed by western blot analysis. (**e**) Representative pictures of normal colon tissues and colon cancer specimens stained with an anti-ELL antibody (upper panel) or anti-c-Myc antibody (lower panel). (**f**,**g**) Expression of c-Myc (**f**) and ELL (**g**) in human colon cancer specimens (*n*=90) and normal colon tissues (*n*=86) as revealed by immunohistochemistry using an anti-c-Myc and anti-ELL antibodies, respectively.

To obtain more insight into the role of ELL in cancer development, we performed xenograft tumour growth assays using three stable HCT116 cell lines described above. After the cells were subcutaneously injected into 3–4-week-old male nude mice (*n*=5 per group), tumour size was measured every week from week 3. The growth rate of HCT116 cell tumours overexpressing the ELL(C595A) mutant was almost the same as that of the control; only one tiny tumour formed after inoculation of HCT116 cells that overexpressed ELL after week 5 (Fig. 8a,b). In addition, no obvious difference in tumour weight between the control and ELL(C595A) tumours (Fig. 8c). Tumour expression of ELL and ELL(C595A) was confirmed (Fig. 8d). These data suggest that ELL inhibits colon cancer xenograft tumour growth.

Cachexia was observed in one mouse with an ELL(C595A)-expressing tumour that died at week 4. In addition, at week 6 when the tumours were harvested, we found that two mice with ELL(C595A)-expressing tumours also exhibited cachexia (Supplementary Fig. 12A, red arrows), but we did not find cachexia symptom in the control mice with similar tumour burden. Because cachexia is one of the common symptoms exhibited in advanced cancer patients^38^, we analysed the tumours of these two mice in detail. The tumours attached much tighter to the ribs. In addition, macro-metastasis to the lung in these two mice was confirmed by histological analysis (Supplementary Fig. 12B–D). These data suggest that the ELL(C595A) mutant not only loses the tumour suppressive function, but also gains a function for promoting tumour metastasis.

To further confirm the above observations, we repeated xenograft tumour growth assays. Due to no obvious tumours were formed in mice with injections of ELL-overexpressing HCT116 cells, we excluded this cell line but added HCT116 parental cells as another control. No significant difference in the size and the growth rate of xenograft tumours was observed among these three groups: parental cells, pHAGE control and ELL(C595A)-overexpressing cells (Supplementary Fig. 13A and B). Similar to the above observation (Supplementary Fig. 12A), cachexia exhibited in two mice with injections of ELL(C595A)-overexpressing HCT116 cells (Supplementary Fig. 13A, red arrows). Particularly, one of the mice (Supplementary Fig. 13A, yellow arrows) not only developed macro-metastasis in the whole chest (Supplementary Fig. 13C), but also exhibited potential bone invasion (Supplementary Fig. 13D). In addition, the other three mice developed micro-metastasis in lung (Supplementary Fig. 13E and F). These data further confirm gain of function of ELL(C595A) mutant in promoting metastasis.

To figure out the mechanisms regarding why the ELL(C595A) mutant promotes tumour metastasis, initially, we performed cell invasion assays. Overexpression of ELL(C595A) could enhance invasive ability of cells (Supplementary Fig. 14). Therefore, ELL(C595A) mutation might cause the cells to gain more invasive capability but not of proliferation rate. Subsequently, we conducted quantitative analysis of global proteome in six xenograft tumours (three pHAGE control tumours versus three pHAGE-ELL(C595A) tumours) (Supplementary Fig. 15A). After the assays by comparing 3 pHAGE-ELL(C595A) tumours with 3 pHAGE control tumours, 11 proteins were identified to be upregulated in pHAGE-ELL(C595A) tumours (up-ratio>1.2, *P*<0.05) (Supplementary Fig. 15B). To confirm these upregulations, we initially performed semi-quantitative RT–PCR assays (Supplementary Table 2). The upregulations of *S100A4*, *MARCKSL1*, *BCAM*, *BAG4*, *IPO4* and *CPSF7* in pHAGE-ELL(C595A) tumours were confirmed (Supplementary Fig. 15C). Then, using a commercially available antibody, anti-S100A4, we validated upregulation of S100A4 in pHAGE-ELL(C595A) tumours (Supplementary Fig. 15D). *S100A4*, *MARCKSL1* and *BAG4* have been reported to play important roles in promoting tumour metastasis^39^,^40^,^41^,^42^,^43^,^44^,^45^,^46^,^47^,^48^. Thus, ELL(C595A) mutant might promote tumour metastasis through inducing these metastasis-associated genes.

We further performed immunohistochemistry (IHC) analysis of human colon cancer arrays. ELL and c-Myc were mainly detected in the nucleus of epithelial cells (Fig. 8e). The frequency of c-Myc-positive nuclear staining increased in colon cancer specimens compared with that in the non-tumour containing tissues (72.2% versus 41.86%, respectively; Fig. 8f)^49^. In contrast, the frequency of ELL-positive staining decreased in colon cancer specimens compared with the non-tumour containing tissues (37.7% versus 65.12%, respectively; Fig. 8g). These data suggest that ELL is downregulated in colon cancer, which is negatively correlated with the elevated expression of c-Myc.

Based on the observations, we propose a working model of ELL-mediated c-Myc degradation (Supplementary Fig. 16).

## Discussion

A series of studies revealed that *ELL* plays important roles in transcription control, particularly as a component of both SEC and LEC^4^,^6^. Nonetheless, in mammals, the *in vivo* physiological function of *ELL* has remained poorly understood in mechanistic terms due to the early embryonic lethality of *ELL*-null mice^12^. In this study, we confirmed the ubiquitin ligase activity of ELL. However, no obvious conserved structural domains of typical E3 ligases, such as a RING-FINGER or a HECT domain^19^, have been identified in ELL. Moreover, it appears that ELL cannot be attributed to any of the components in the multiple RING-type E3 complexes^19^. Therefore, ELL might represent a novel type of E3 ubiquitin ligase. Given that ELL(C595A) mutant is inactive for catalysing poly-ubiquitination, it appears that ELL has characteristics similar to that of HECT and RBR-domain E3 ligases, in which an active cysteine is required for accepting ubiquitin from the E2 and then transferring it from the E3 to the substrate^19^.

Regarding that plenty of studies strongly support a crucial role for *ELL* in the control of transcriptional elongation, it is possible that these effects may be mediated through targeting other components of either the SEC or LEC complex for proteasomal degradation. In fact, some E3 ligases regulate transcription elongation through targeting their substrates^50^,^51^,^52^. Thus, no irreconcilable contradictions exist between the identification of *ELL* as an E3 ligase and its recognized role as a key regulator of transcriptional elongation. Moreover, because c-Myc has also been shown to regulate transcription elongation^53^, ELL may regulate transcription elongation through modulating c-Myc.

E3 ligase usually has multiple targets. Thus, ELL might also have other targets in addition to c-Myc. Recent work showed that ELL interacts with the THIIF complex and that helps the RNA polymerase II to restart the transcription after DNA repair^16^. ELL E3 ubiquitin ligase activity can nicely explain this function of ELL^16^. In addition, ELL was also previously purified as a component in combined nuclear and cytoplasmic lysates that were bound to ELL-associate proteins (EAP), including EAP45, EAF30 and EAF20 (ref. 54). These proteins were later found to be identical to Vps 36, Vps 22 and Vps 25 of the endosomal sorting complex required for transport system (ESCRTII)^55^. The ESCRT system is linked to degradation of growth factor receptors like epidermal growth factor receptor (EGFR)^56^. Therefore, ELL E3 ligase might also involve in transcription regulation by targeting membrane bound growth factor receptors for degradation through the ESCRT II pathways.

Of note, the oncogenic fusion protein, MLL-ELL, which contains aa 46–621 of ELL^1^, loses the ability to promote c-Myc poly-ubiquitination and degradation. This phenomenon might be a result of a conformation change in ELL after its fusion with MLL^13^,^15^. To date, the mechanism by which MLL-ELL induces AML remains poorly understood^57^. It is possible that loss of the ability to induce c-Myc degradation might underlie MLL-ELL-related leukaemogenesis. In the future, it is worth to collect the AML samples with MLL-ELL translocation and check them to see whether c-Myc protein in these samples is stabilized.

It seems that the protein level of c-Myc protein varied among ELL(C595A) mutant xenograft tumours (Fig. 8d; Supplementary Fig. 13A). Based on protein degradation and ubiquitination assays *in vitro* and *in vivo*, ELL(C595A) mutant lost its E3 ligase activity. So, it was supposed not to affect c-Myc protein level. However, regarding that ELL(C595A) mutant could still bind to ELL and suppress *E2F2* expression, ELL(C595A) mutant might still regulate the activity or the stability of c-Myc through an yet-unknown mechanism other than acting E3 ligase activity.

Factors that modulate c-Myc activity might contribute to tumour initiation and progression^18^. *Fbw7* is a well-defined tumour suppressor as it downregulates *c-Myc* activity^18^. In contrast, *Skp2* and *HectH9* are considered oncogenes because they positively regulate c-Myc transcriptional activity^18^. In this study, we found that ELL suppresses the expression of *hTERT* and *E2F2*, two well-defined c-Myc down-stream target genes, by targeting c-Myc degradation. However, MLL-ELL was unable to suppress *hTERT* and *E2F2* expression. Furthermore, overexpression of ELL in HCT116 colon cancer cells inhibited cell proliferation and xenograft tumour growth in nude mice. In addition, ELL expression was decreased in human colon cancer specimens compared with normal tissues, and was negatively correlated with c-Myc expression. These observations suggest that ELL might contribute to tumour suppression.

In this study, we provided evidences to show that suppression of *hTERT* and *E2F2* expression by ELL is dependent on c-Myc (Supplementary Fig. 8). In addition, it appears that the inhibitory role of ELL on cell proliferation is also mediated by c-Myc regarding the facts that overexpression of ELL inhibited Rat1 cell (wild-type c-Myc) proliferation, but did not inhibit HO15.19 cell (c-Myc deficient) proliferation, and that overexpression/knockdown of ELL in HCT116 cells inhibited or enhanced cell proliferation with a good correlation of endogenous c-Myc level (Fig. 7). Given that overexpression of ELL inhibited xenograft tumour growth and ELL expression was negatively correlated with c-Myc expression in human colon cancer specimens, the suppressive role of ELL on tumour growth might also be mediated by c-Myc. However, in this study, we could not provide direct evidence to support this statement (Fig. 8). In fact, we have tried to get direct evidence to confirm the c-Myc-dependent tumour suppressive role of ELL through establishing stable cell lines using three-round lentivirus infections based on the established stable cell lines with endogenous c-Myc knockdown plus overexpression of wild-type c-Myc (c-Myc-shRNA resistant) or c-Myc(4K/R) mutant. Unfortunately, when these cell lines went through the third-round infection with the lentiviruses expressing ELL or ELL(C595A) mutant as well as ELL-shRNA, the cells became very unhealthy (the most of cells were broken) for unknown reasons. In the future, to avoid three-round lentivirus infections, other approaches, such as CRISPR/Cas9, could be employed to knockout of endogenous c-Myc at first and then re-express wild-type c-Myc or c-Myc(4K/R) mutant plus ELL or ELL(C595A) mutant as well as ELL-shRNA for establishing stable cell lines. The xenograft tumour growth assays based on these cell lines might help to resolve this puzzle eventually. Clearly, we still cannot rule out that additional ELL substrates, except for c-Myc, play roles in mediating ELL's function in tumour suppression. Further studies are required to address the identity and function of such substrates.

Although the enzymatic inactivated mutant, ELL(C595A), lost its ability to suppress *hTERT* expression, it did inhibit *E2F2* expression. ELL(C595A) also lost its ability to inhibit cancer cell proliferation and xenograft tumour growth. *hTERT* is considered to be an oncogene, mediating the oncogenic function of c-Myc^58^. In contrast, the role of *E2F2*, as well as its family members, such as *E2F1*, on tumorigenesis is varied from case to case, serving either as oncogene or a tumour suppressor^59^. Indeed, *E2F2* is reported to suppress c-Myc-induced proliferation and tumorigenesis^60^. Therefore, it appears that the E3 ubiquitin ligase activity of ELL is particularly required for its tumour suppressive function.

Notably, the ELL(C595A) mutant not only lost its ability to suppress cell proliferation and xenograft tumour growth, but also promoted metastasis, likely by gaining an invasive capability, which is similar to that exhibited by classic tumour suppressors, such as *p53* and *pVHL*^61^,^62^. The mechanistic studies via quantitative analysis of global proteome in xenograft tumours revealed that some metastasis-associated proteins, including S100A4, MARCKSL1 and BAG4 were increased in ELL(C595A) xenograft tumours compared to the control tumours. S100A4 is a calcium-binding protein with metastasis-promoting function^40^,^63^,^64^, which can induce motility and invasion of glioblastoma cells^39^, participates in epithelial–mesenchymal transition in breast cancer, and involves in liver metastasis of colorectal cancer^65^. In addition, silencing of *S100A4* via siRNA or shRNA and blocking of S100A4 via anti-S100A4 antibody can reduce metastasis formation by blocking stroma cell invasion^41^,^42^,^43^. *MARCKSL1* exhibits anti-angiogenic effects through suppression of VEGFR-2-dependent Akt/PDK-1/mTOR phosphorylation^45^. *BAG4* is a negative regulator of apoptosis, which also links to tumour aggressiveness and metastasis^47^,^48^. Therefore, ELL(C595A) mutant might promote tumour metastasis through inducing expression of metastasis-associated genes, particularly for inducing *S100A4*, a well-defined metastasis-promoting gene. However, how and why ELL(C595A) mutant induces expression of these genes is still unknown. To further investigate this induction and its contribution to tumour metastasis will give insight into the physiological function of ELL(C595A) mutant and the underlying mechanisms, leading to new treatments for tumour metastasis.

## Methods

### Cell culture and transfection

HEK293, HEK293T, HCT116 and Cos7 cells were originally obtained from ATCC. Rat1 and HO15.19 cells (c-Myc null rat fibroblast Rat1 cells) were obtained from Dr Guoliang Qing's lab. HEK293, HEK293T, Cos7, Rat1 and HO15.19 cells were maintained in Dulbecco's modified Eagle's medium (HyClone) and HCT116 cells were maintained in McCoy's 5A medium (HyClone), supplemented with 10% fetal bovine serum (FBS; Hyclone) at 37 °C in a humidified atmosphere incubator containing 5% CO_2_. All cell lines were verified to be free of *Mycoplasma* contamination before use. VigoFect (Vigorous Biotechnology) was used for cell transfection.

### Antibodies and reagents

The antibodies used were as follows: anti-ELL antibody (A0668, 1:1,000 for IB analysis, ABclonal; 51044-1-AP, 1:1,000 for IB analysis, Proteintech; HPA046076, 1:200 for IHC staining, Sigma-Aldrich), anti-c-Myc antibody (9E10, 1:1,000 for IB analysis, 1:200 for IHC staining, Santa Cruz; A0309, 1:100 for endogenous IP analysis, ABclonal; D84C12, 1:1,000 for IB analysis, Cell Signaling), anti-Flag antibody (F1804, 1:1,000 for IB analysis, Sigma); anti-HA antibody (1:5,000 for IB analysis, Covance), anti-His antibody (H15, 1:1,000 for IB analysis, Santa Cruz), anti-GAPDH antibody (SC-47724, 1:1,000 for IB analysis, Santa Cruz), anti-S100A4 (ab27957, 1:1,000 for IB analysis, Abcam) and anti-α-tubulin antibody (EPR1333, 1:10,000 for IB analysis, Epitomics). The reagents used were as follows: chloroquine diphosphate (BioVison), AICAR (Cayman), MG132 (Calbiochem), cycloheximide (Sigma-Aldrich).

### Plasmid constructs and mutants

The original wild-type *c-Myc* and its domain constructs were kindly provided by Stephen Hann. HA-tagged c-Myc (P57S) and c-Myc (T58A) were kindly provided by Scott Lowe. The original *ELL* and *MLL-ELL* constructs were kindly provided by Ali Shilatifard. The *hTERT* promoter luciferase reporter was kindly provided by Tae Kook Kim. The GFP-BM5 construct was kindly provided by Masayuki Komada.

The domain constructs of ELL, His-ELL, Flag-ELL, pcDNA-ELL and GFP-ELL have been described previously^15^. pSuper-ELL-shRNA-1 and pSuper-ELL-shRNA-2 were constructed in the pSuper vector using the following targeting sequences: 5′-CAACACCAACTACAGCCAGGA-3′ (ELL-shRNA-1) and 5′-GCGAGTACCTGCACAGCAA-3′(ELL-shRNA-2). His-ubiquitin and His-ubiquitin (K48R) have been described previously^66^. The ELL(C595A) mutant was generated by PCR. The human c-Myc mutants, including S62A, S62E, K51/52R, K126R, K144/149R, K158R, K206R, K269R, K275R, K289R, K298R, K317/323R, K323R, K326R, K342R, K355R, K371R, K389R, K392R, K397R, K398R, K412R, K422R, K428R, K430R and 4K/R(K51/51/397/430R), were generated by PCR and cloned into the pCGN-HAM vector (kindly provided by William Tansey). The *E2F2* promoter luciferase reporter (E2F2-luc.) was generated by PCR and cloned into pGL3-Basic (Promega). PM-c-Myc was generated by PCR and cloned into PM vector (which contains GAL4-binding domain; Clontech).

The human E2 ubiquitin-conjugating enzyme expression plasmids, including *UbcH1*, *UbcH2*, *UbcH3*, *UbcH5a*, *UbcH5b*, *UbcH5c*, *UbcH6*, *UbcH7*, *UbcH8*, *UbcH10* and *Ubc13*, were generated by PCR using the indicated primers (Supplementary Table 1), and cloned into CMV-Myc vector (Clontech).

### Co-immunoprecipitation and western blot analysis

Anti-c-Myc antibody, anti-HA antibody and anti-Flag antibody-conjugated agarose beads were purchased from Sigma-Aldrich. Protein A/G Sepharose beads were purchased from GE Company. GST-Bind Resin was purchased from Novogen. For western blot analysis and co-immunoprecipitation of overexpressed proteins, the experimental procedures have been described^67^. Because overexpression of ELL caused c-Myc protein degradation, thus, we transfected 2–3 times more *c-Myc* expression plasmid (HA-c-Myc) when co-transfecting with *ELL* expression vector compared with co-transfection with *c-Myc* expression vector and the empty vector control. For endogenous co-immunoprecipitation, the experimental procedures have been described previously^15^. For GST pull-down assays, GST-tagged c-Myc and His-tagged ELL were expressed in *E. coli* (BL21) and purified. After co-immunoprecipitation using GST-Bind Resin, the protein was separated by SDS–polyacrylamide gel electrophoresis. The gel was stained with Coomassie blue or transferred to a polyvinylidene difluoride membrane for detecting His-ELL by western blot analysis. The Fuji Film LAS4000 mini luminescent image analyzer was used for photographing the blots. Multi Gauge V3.0 was used for quantifying the protein levels based on the band density obtained in western blot analysis. The full-size images are shown in Supplementary Fig. 17.

### *In vitro* and *in vivo* ubiquitination assays

*In vitro* ubiquitination was performed according to the protocol provided by the Ubiquitination Kit (UW9920, BioMol) with some modifications. Briefly, His6-c-Myc, His6-ELL and His6-ELL(C595A) were expressed in *E. coli* and purified by Ni^2+^-NTA resin (Novagen). The assays were carried out at 37 °C in a 50-μl reaction mixture containing 20 U ml^−1^ of inorganic pyrophophatase (Sigma-Aldrich), 5 mM dithiothreitol, 5 mM Mg-ATP, 100 nM E1, 2.5 μM indicated E2, 0.75–1 mM E3 (1 mM His-ELL or 0.75 mM His-ELL(C595A)), 1 μM target protein (His6-c-Myc) and 2.5 μM biotin-labelled ubiquitin. After incubated for 30–60 min, the reactions were quenched by addition of 50 μl of 2 × non-reducing gel-loading buffer and separated using 12% SDS–polyacrylamide gel electrophoresis. To get accurate results, the PAGE gel was run for a relatively longer time until protein bands smaller than 40 kDa ran out of the bottom line of the gel as judged by the protein molecular weight marker. Then the protein was transferred to a polyvinylidene difluoride membrane. To reduce the background, anti-c-Myc antibody (A0309, 1:1,000, ABclonal) was used for detecting poly-ubiquitination of c-Myc via western blot analysis instead of using HRP-Streptavidin detection system recommended by the kit for detecting biotinylated-ubiquitin.

For *in vivo* ubiquitination assays, HEK293T cells were co-transfected with HA-c-Myc, Myc-ELL, Myc-ELL(C595A), His-ubiquitin or His-ubiquitin (K48R). Ubiquitination assays with His-ubiquitin or His-ubiquitin (K48R) were performed by affinity purification on Ni^2+^-NTA resin (Novagen). An anti-HA antibody was used for detecting c-Myc poly-ubiquitination.

### Lentivirus-mediated gene overexpression and knockdown

ELL, ELL(C595A), c-Myc and c-Myc (4K/R) were subcloned into the lentivirus vector pHAGE-CMV-MCS-IZsGreen. c-Myc short hairpin RNA (shRNA) was cloned into the lentivirus vector LentiLox3.7 with the following targeting sequence: 5′-GCCATAATGTAAACTGCCT-3′ (which targets 5′ untranslated region of human *c-Myc*). ELL short hairpin RNA was cloned into the lentivirus vector pLKO.1 vector with the following targeting sequence: 5′-CAACACCAACTACAGCCAGGA-3′.

Lentiviruses for gene overexpression were generated by transfecting HEK293T cells with a transducing vector, and the packaging vectors, psPAX2 and pMD2.G. Lentiviruses for *c-Myc* knockdown or scrambled control were generated by transfecting HEK293T cells with a transducing vector or a control vector, and the packaging vectors, VSVG, pRSV-Rev and pMDLg/pRRE. Lentiviruses for ELL knockdown or scrambled control were generated by transfecting HEK293T cells with a transducing vector or a control vector, and the packaging vectors, psPAX2 and pMD2.G.

After transfection for 8 h, the medium was replaced with fresh Dulbecco's modified Eagle's medium with 10% FBS. After 40 h, virus particles in the medium were collected, filtered and transduced into target cells. Polybrene (8 μg ml^−1^) was added to the medium to improve infection efficiency.

### Luciferase reporter assays

Cells were seeded in 24-well plates and transfected with the indicated luciferase reporters using VigoFect (Vigorous Biotechnology). pTK-*Renilla* was used as an internal control. For mammalian one-hybridization assays, the pRF-luciferase construct (Stratagene) was used as reporter. Luciferase activity was measured 20–24 h after transfection using the Dual-luciferase Reporter Assay System (Promega).

### Semi-quantitative RT–PCR

Total RNA was extracted from cells using the Trizol reagent (Invitrogen), and cDNA was synthesized using a first strand cDNA synthesis kit (Fermentas). The following primers were used for RT–PCR analysis: human *c-Myc*, sense: 5′-TTCTGTGGAAAAGAGGCAGG-3′ and antisense: 5′-TGCGTAGTTGTGCTGATGTG-3′; human *TERT*, sense: 5′-CGGAAGAGTGTCTGGAGCAA-3′ and antisense: 5′-GGATGAAGCGGAGTCTGGA-3′; human *E2F2*, sense: 5′-GGCCAAGAACAACATCCAGT-3′ and antisense: 5′-TGTCCTCAGTCAGGTGCTTG-3′. The 18 s RNA was used as an internal control: sense: 5′-TCAACTTCGATGGTAGTCGCCGT-3′ and antisense: 5′-TCCTTGGATGTGGTAGCCGTTCT-3′.

### Nucleus and nucleolus localization assays

Cos7, HEK293T and HCT116 and Cos7 cells were co-transfected with the indicated plasmids respectively. The plasmids include GFP-tagged BM5 (GFP-BM5), RFP-tagged c-Myc (RFP-c-Myc), GFP-tagged ELL (GFP-ELL), RFP-tagged Max (RFP-Max), RFP-tagged Mxd (RFP-Mxd), HA empty vector, HA-ELL and HA-c-Myc. After transfected for 16–24 h, the cells were examined under a fluorescence microscope (Nikon) or a co-focal microscope (Zeiss).

### ChIP assays

The ChIP assays were performed according to the protocol described previously^15^ with modification. Briefly, HCT116 cells with indicated transfections for ELL overexpression or knockdown were fixed in 1% formaldehyde and then lysed in SDS buffer. Lysates were sonicated yielding DNA fragments with an average size of 200–1,000 bp and precleared with protein A/G agarose beads. Then lysates were immunoprecipitated by 5 μg of anti-c-Myc antibody (A0309, ABclonal), or normal rabbit immunoglobulin-G. Antibody-nucleoprotein complex mixtures were incubated overnight and recovered by incubation with 20 μl of protein A/G agarose beads. After incubation, the protein A/G beads were washed and eluted. The eluted solutions were used for detecting the promoter region of *hTERT* or *E2F2* by semi-quantitative RT–PCR assays. The DNA level detected in the sample with immunoprecipitation by anti-c-Myc antibody was normalized to the sample with immunoprecipitation by rabbit immunoglobulin-G control. The primers specific for the *hTERT* promoter region are 5′-TCCCCTTCAGTCCGGCATT-3′(forward) and 5′-AGCGGAGAGAGGTCGAATCG-3′(reverse). The primers specific for the *E2F2* promoter region are 5′-AAGTCGGTGCAGTCGAGACC-3′(forward) and 5′-GAGATCGCCGCTTGGAGATCG-3′(reverse).

### *In vitro* cell growth assays

After HCT 116 cells were stably transfected with ELL, ELL(C595A), empty vector control, ELL-shRNA or scrambled control via lentivirus infection, they were seeded in six-well plates at 1 × 10^5^ cells per well and counted at days 1, 2, 3 and 4 using an automatic cell counter (TC10; Bio-Rad).

Similarly, Rat1 and HO15.19 cells were stably transfected with ELL, or empty vector control via lentivirus infection, they were seeded in six-well plates at 1 × 10^5^ cells per well and counted at days 1, 2, 3 and 4 using an automatic cell counter (TC10; Bio-Rad).

### Colony formation assays

The stable-transfected HCT116 cells were seeded in six-well plates at 2 × 10^4^ cells per well (for ELL overexpression) or 1.5 × 10^4^ cells per well (for ELL knockdown). After 6 days, the colonies were fixed using methanol and stained with crystal violet (0.5% in methanol). The colony numbers were counted based on the images obtained by a stereo microscope. Colonies of a suitable size were counted and the numbers counted from the same area were used for comparison.

### Cell invasion assays

Transwell plates were purchased from Corning (Costar 3422). Cell invasion assays were performed following the protocol provided by the manufacturer. Briefly, HCT116 cells with different lentivirus infections were suspended in serum-free McCoy's 5A medium and put into the top wells at 5 × 10^5^ cells per well; McCoy's 5A medium supplemented with 10% FBS was added into the bottom wells. After cultured for 36 h in a humidified atmosphere incubator containing 5% CO_2_, the wells were taken out, then fixed and stained by Giemsa. The pictures were taken under a Leica stereo microscope and the colony numbers were counted by ImageJ software.

### Immunohistochemical analysis

Colon cancer tissue arrays were obtained from Shanghai Zuocheng Bio. Co., Ltd (cat. no. HCol-Ade180Sur-04). Immunohistochemical staining was also provided by the company. This array contained 180 tissues, including 90 colon cancer specimens and 90 normal colon tissues isolated from patients with colon cancer. For comparison, two sequential arrays were obtained for the immunohistochemical staining of c-Myc and ELL using a monoclonal antibody against c-Myc (9E10, 1:500 for IHC staining, Santa Cruz) and a polyclonal antibody against ELL (HPA046076, 1:500 for IHC staining, Sigma-Aldrich). After staining, four normal colon tissues were lost in the array, so only 86 normal colon tissues were counted for further data analysis. Immunostaining was evaluated manually and graded using a two-score system based on intensity score and proportion score described previously^68^. Samples with an intensity score >1.5 and a proportion score >50% were considered to have positive staining.

### *In vivo* tumour growth in xenograft models

Animal studies were approved by the Animal Care and Use Committee of Institute of Hydrobiology, Chinese Academy of Sciences. HCT116 cells were infected with lentivirus encoding ELL, ELL(C595A) or empty vector control. For the first experiment, 15 male nude mice (3–4 weeks of age) were randomly separated into three groups (*n*=5 per group) and each mouse was injected subcutaneously in the flank region with 2 × 10^6^ infected HCT116 cells. Three groups were injected with the three different cell lines, including pHAGE-ELL, pHAGE-ELL(C595A) and pHAGE control, respectively. For the second experiment, 15 male nude mice (4–5 weeks of age) were also randomly separated into three groups (*n*=5 per group) and each mouse was injected subcutaneously in the flank region with 2.2 × 10^6^ parental or infected HCT116 cells. Three groups were injected with the three different cell lines, including parental HCT116 cells, pHAGE control and pHAGE-ELL(C595A), respectively. The tumour volume was measured weekly starting at week 3 using the following formula: *V*=*π*.*abc*/6 (ref. 69). After 6 weeks, the mice were killed and the tumours were harvested to determine their weight and for gene expression analysis. For detecting metastasis, the lungs were also harvested and histological analysis was carried out after hematoxylin and eosin staining. All animal protocols were approved by the Institutional Animal Care and Use Committee of Institute of Hydrobiology, Chinese Academy of Sciences.

### Quantitative analysis of global proteome in xenograft tumours

Ten male nude mice (4–5 weeks of age) were randomly separated into two groups (*n*=5 per group) and each mouse was injected subcutaneously in the flank region with 2.2 × 10^6^ HCT116 cells infected with pHAGE control or pHAGE-ELL(C595A). After 3 weeks, the mice were killed, the tumours were harvested, and three pHAGE control tumours and three pHAGE-ELL(C595A) tumours were sent to PTM-Biolabs Co., Ltd (Hangzhou, China) for quantitative analysis of global proteome. Semi-quantitative RT–PCR and western blot were employed for verification of gene upregulation. The primers used for semi-quantitative RT–PCR are listed in Supplementary Table 2.

### Statistical analysis

Luciferase, semi-quantitative RT–PCR, *in vitro* cell growth, colony formation and cell invasion assay data are reported as mean ±s.e.m. of three independent experiments performed in triplicate. For the *in vivo* studies, data are reported as mean ±s.e.m. The statistical analysis was performed using GraphPad Prism 5 (unpaired *t*-test) (GraphPad Software Inc.).

## Additional information

**How to cite this article:** Chen, Y. *et al*. ELL targets c-Myc for proteasomal degradation and suppresses tumour growth. *Nat. Commun.* 7:11057 doi: 10.1038/ncomms11057 (2016).

## Supplementary Material

Supplementary Information Supplementary Figures 1-17 and Supplementary Tables 1-2.
