# Author Correction: ELL targets c-Myc for proteasomal degradation and suppresses tumour growth

**DOI:** 10.1038/s41467-023-38151-y

**Published:** 2023-05-23

**Authors:** Yu Chen, Chi Zhou, Wei Ji, Zhichao Mei, Bo Hu, Wei Zhang, Dawei Zhang, Jing Wang, Xing Liu, Gang Ouyang, Jiangang Zhou, Wuhan Xiao

**Affiliations:** 1 The Key Laboratory of Aquatic Biodiversity and Conservation, Institute of Hydrobiology, Chinese Academy of Sciences, 430072 Wuhan, China; 2 State Key Laboratory of Freshwater Ecology and Biotechnology, Institute of Hydrobiology, Chinese Academy of Sciences, 430072 Wuhan, China

Correction to: *Nature Communications* 10.1038/ncomms11057, published online 24 March 2016

This Article contains errors in Figs. 6, 7 and Supplementary Fig. 13.

In Fig. 6d, the Y axis is incorrectly labelled ‘Relative hTERT’. The correct label should read ‘Relative E2F2 mRNA level’.

The incorrect version Fig 6d is shown below.


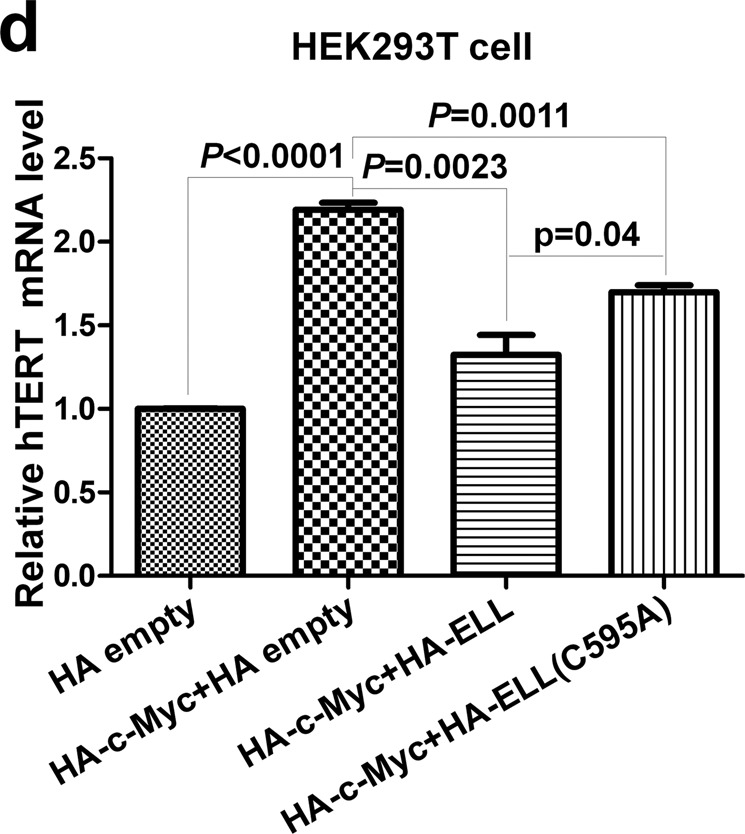


The correct version of Fig. 6d is shown below.


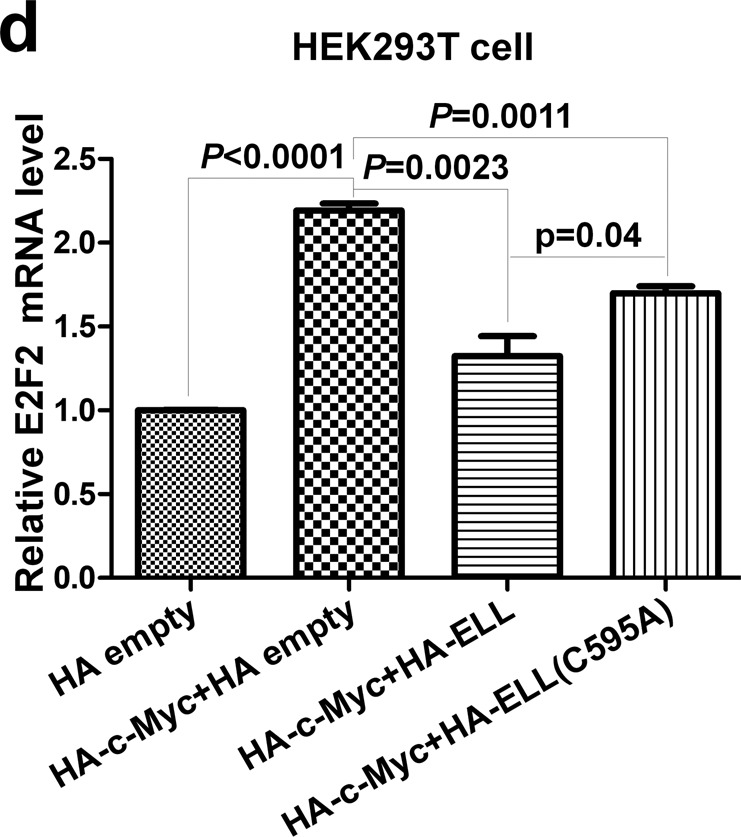


In Fig. 7b, the middle image of the pHAGE control images is a shifted field of view of the first image of the pHAGE control images. In addition, in Fig. 7b, the third image of pHAGE-ELL cells is a shifted field of view image of the first pHAGE-ELL cells image.

The incorrect version Fig 7b is shown below.


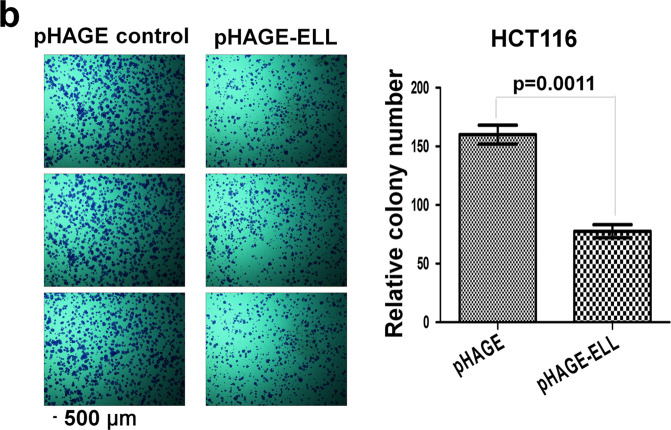


The correct version of Fig. 7b is shown below.


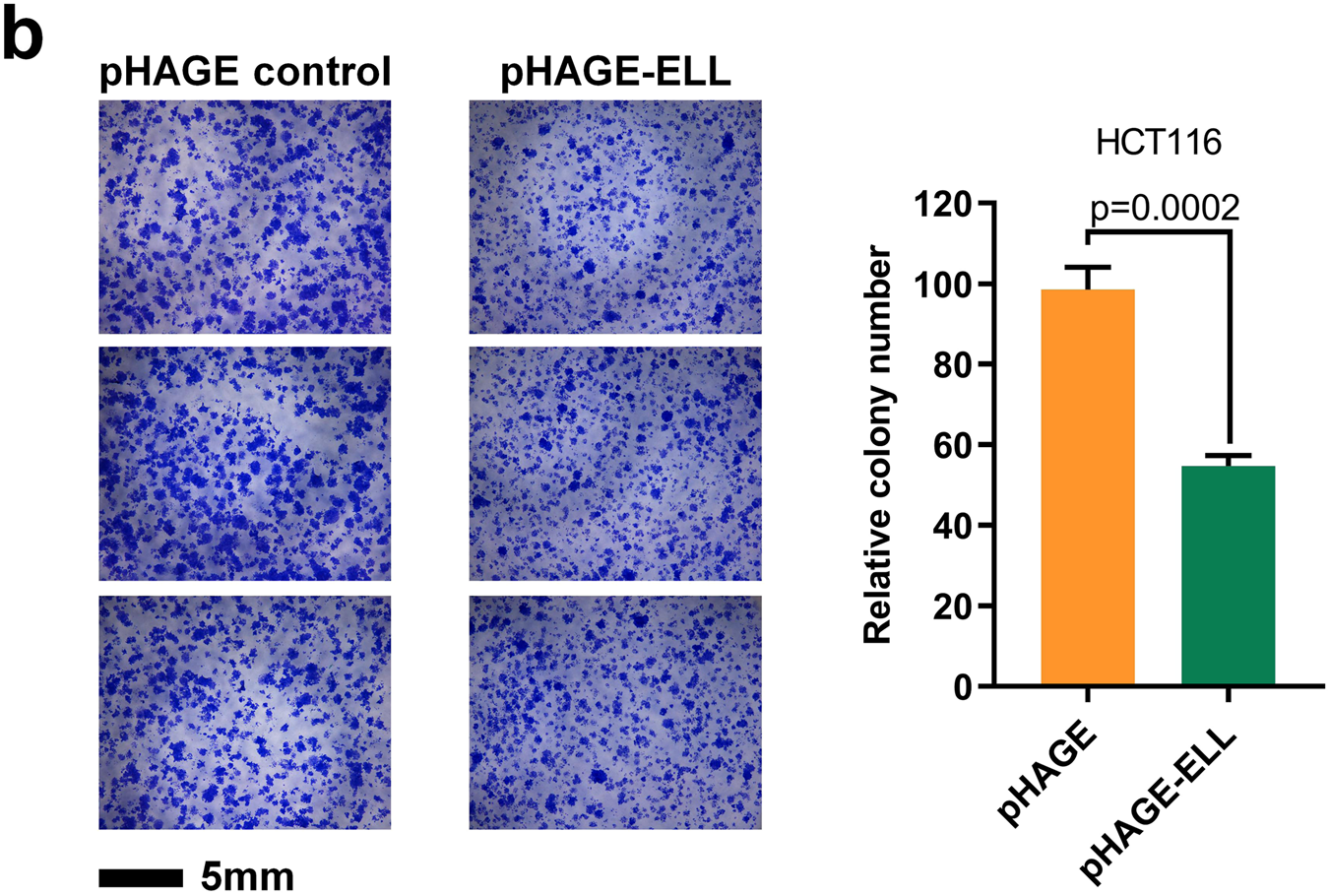


In Fig, 7e, the third image of the pHAGE control images is a shift field of the second image of the pHAGE control cells. In addition, In Fig. 7e, the third image of pHAGE-ELL (C595A) cells is a shifted field of view image of the first pHAGE-ELL (C595A) cells image.

The incorrect version of Fig 7e is shown below.


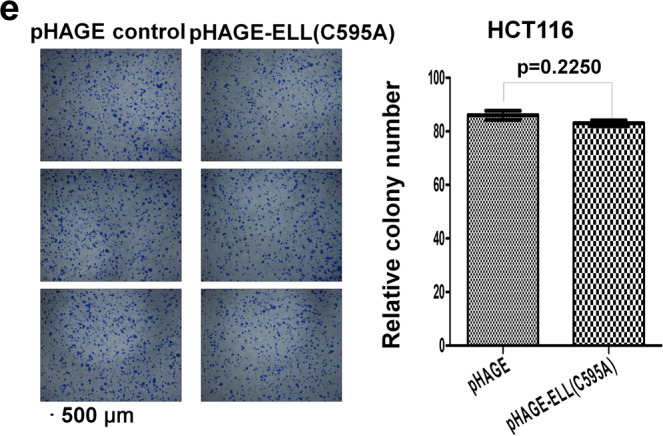


The correct version of Fig. 7e is shown below.


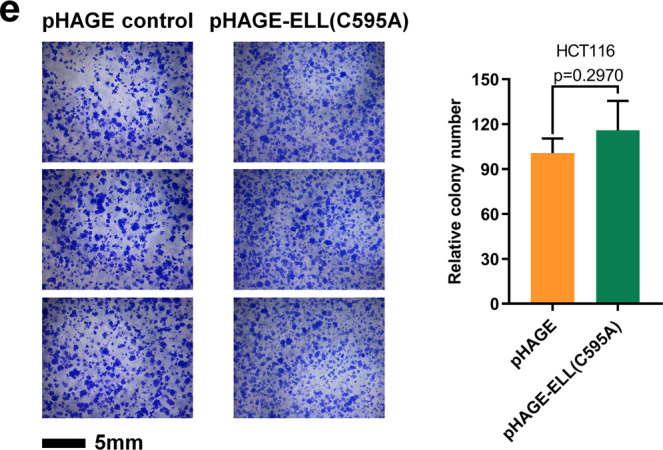


In Fig. 7h, the second and third images for the ELL-shRNA cells are a shifted field of view of the first image for the ELL-shRNA cells.

The incorrect version of Fig. 7h is shown below.


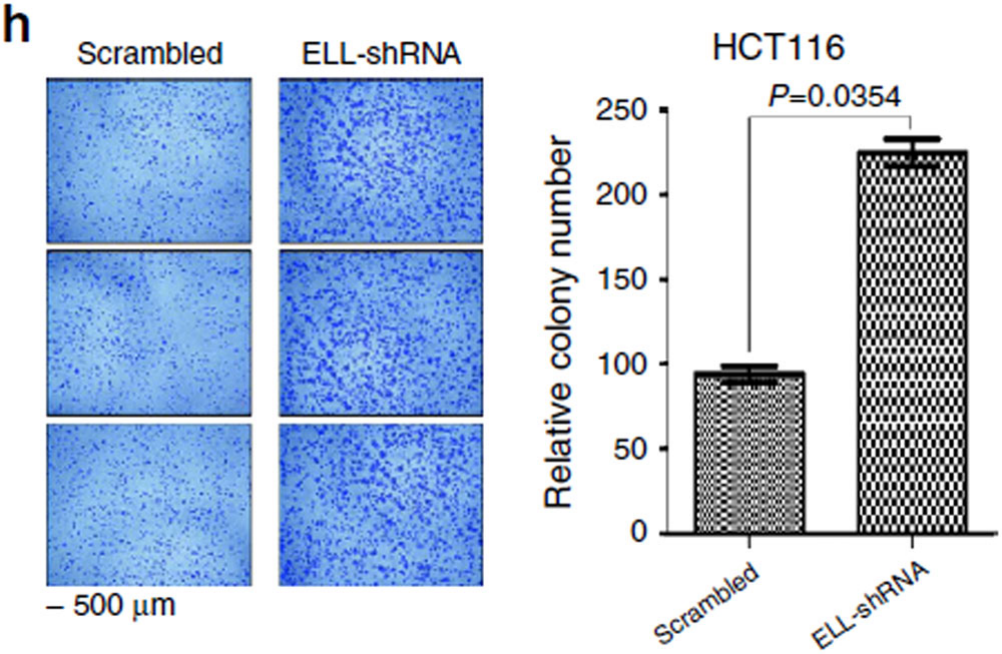


The correct version of Fig. 7h is shown below.


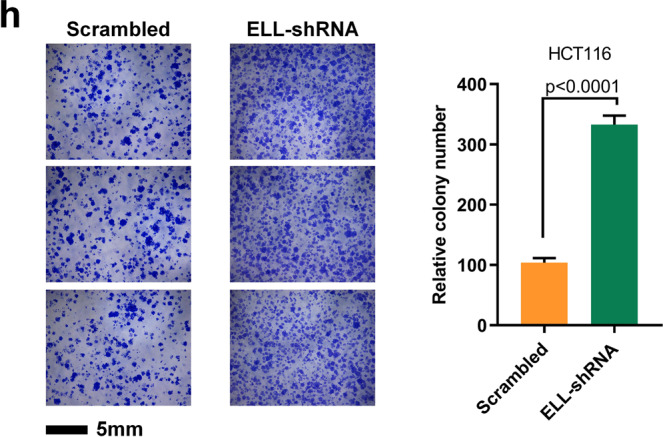


Only two images could be retrieved for Fig. 7b and Fig. 7h, therefore the authors have repeated the experiments three additional times. The raw data is attached as a Supplementary Information file.

In Supplementary Fig. 13e, the top left image incorrectly showed the scale bar as 100µm, this should have been 200 µm. The incorrect version of Supplementary Fig. 13e is shown below.


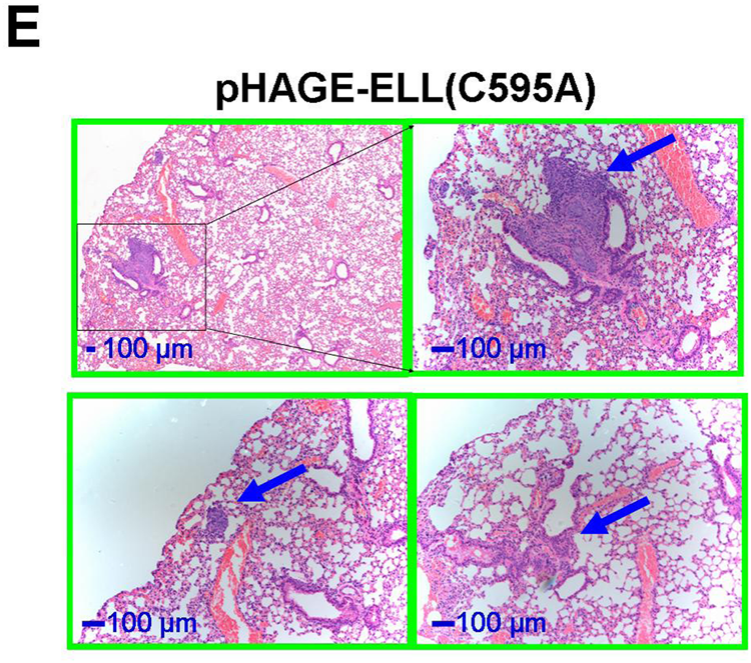


The correct version of Supplementary Fig. 13e is shown below.


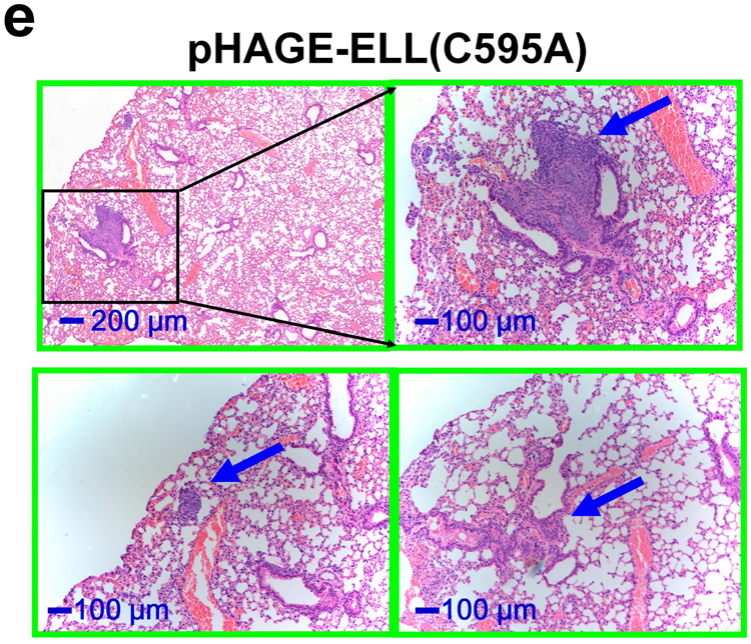


These errors have not been corrected in the PDF or HTML versions of the Article. An updated Supplementary Information file is now provided.

## Supplementary information

Updated Supplementary Information

