# Supplementary material for: Author Correction: ELL targets c-Myc for proteasomal degradation and suppresses tumour growth
Source: Nat Commun. 2023 May 23;14:2972. doi: 10.1038/s41467-023-38151-y (PMC10205805; doi:10.1038/s41467-023-38151-y)
Supplement: Supplementary file 1 — Updated Supplementary Information [file 41467_2023_38151_MOESM1_ESM.pdf]

Supplementary Figure 1

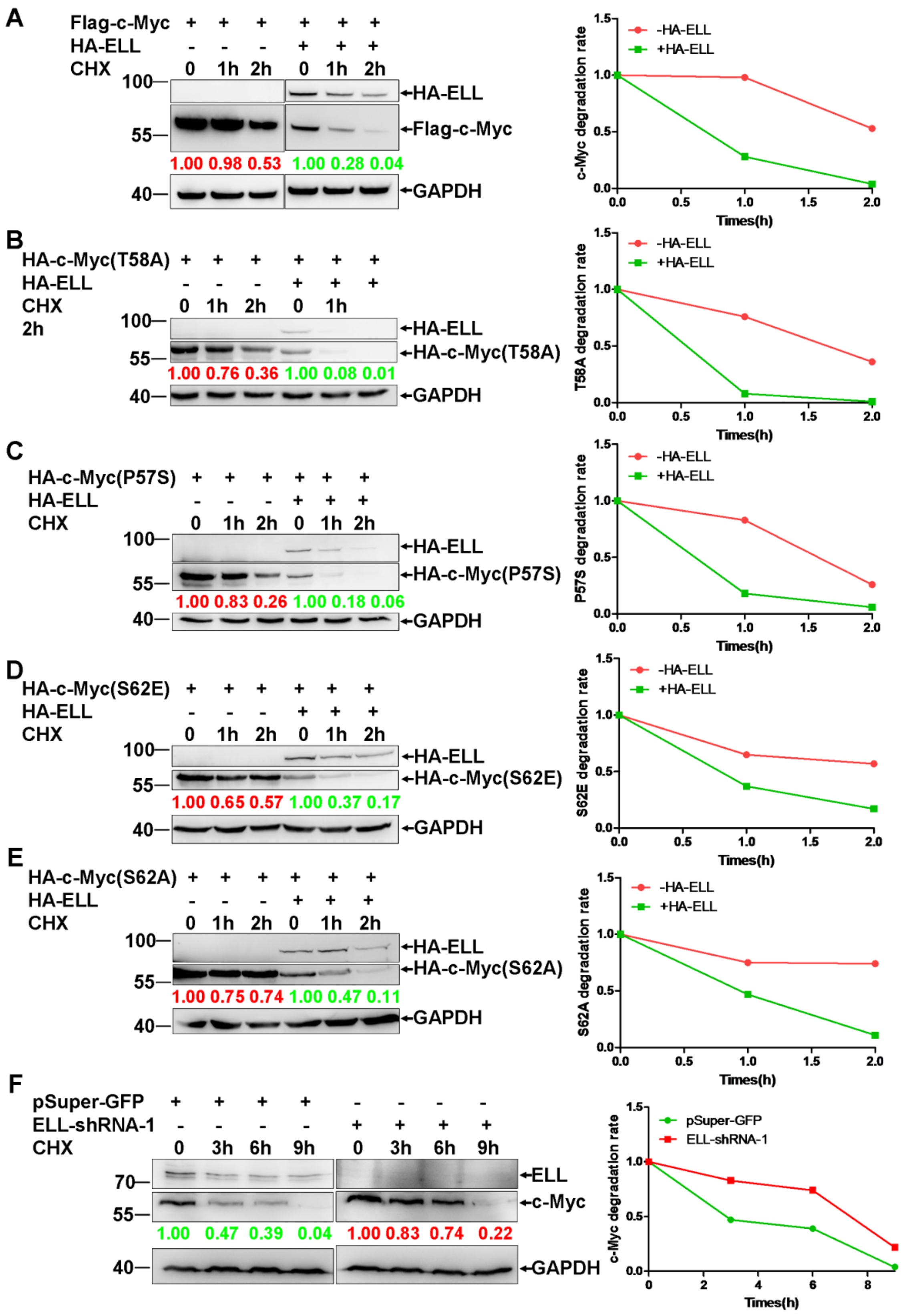

Supplementary Fig. 1. ELL Induces c-Myc Degradation. (A) Overexpression of HA-ELL in HEK293 cells promotes Flag-c-Myc degradation in the presence of cycloheximide (CHX, 50μg/mL). (B-E) Overexpression of HA-*ELL* in HEK293 cells promotes degradation of c-Myc mutants, HA-c-Myc(T58A) (B), HA-c-Myc(P57S) (C), HA-c-Myc(S62E) (D), and HA-c-Myc(S62A) (E), in the presence of cycloheximide (CHX, 50μg/mL). (F) Knockdown of ELL in HCT116 cells via ELL-shRNA-1 enhances the stability of endogenous c-Myc protein level in the presence of cycloheximide (CHX, 50μg/mL).

Supplementary Figure 2

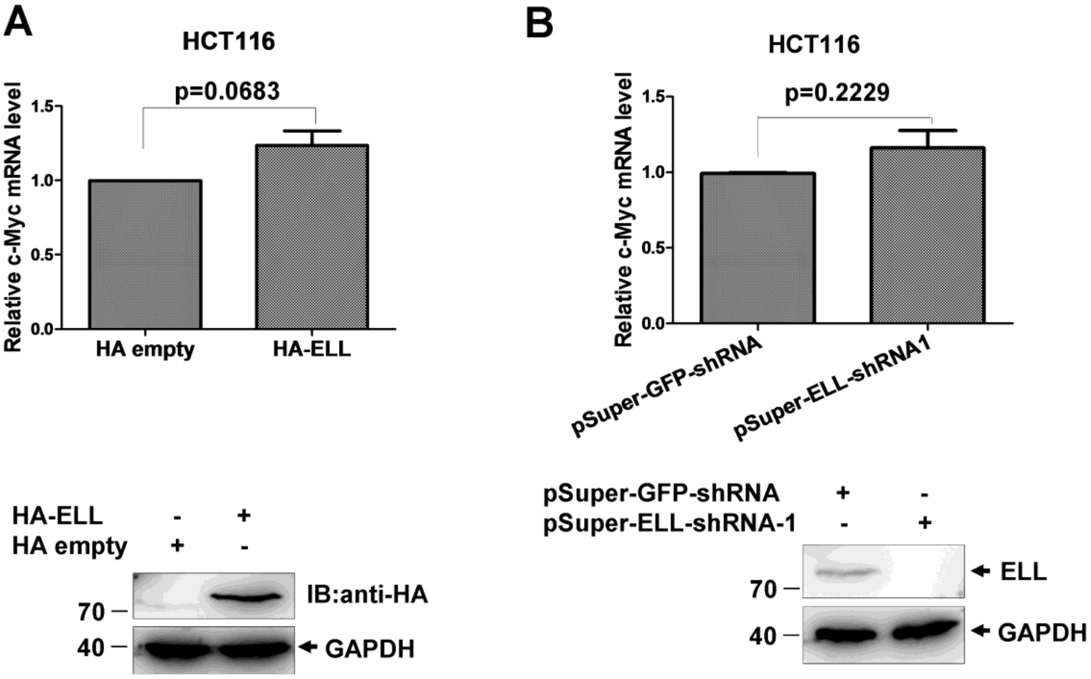

Supplementary Fig. 2. ELL has no obvious effect on the transcription of *c-Myc*. (A) Overexpression of ELL in HCT116 cells does not change *c-Myc* mRNA level as revealed by semi-quantitative RT-PCR assays (p=0.0683, *t*-test). (B) Knockdown of ELL in HCT116 cells does not change *c-Myc* mRNA level as revealed by semi-quantitative RT-PCR assays (p=0.2229, *t*-test).

Supplementary Figure 3

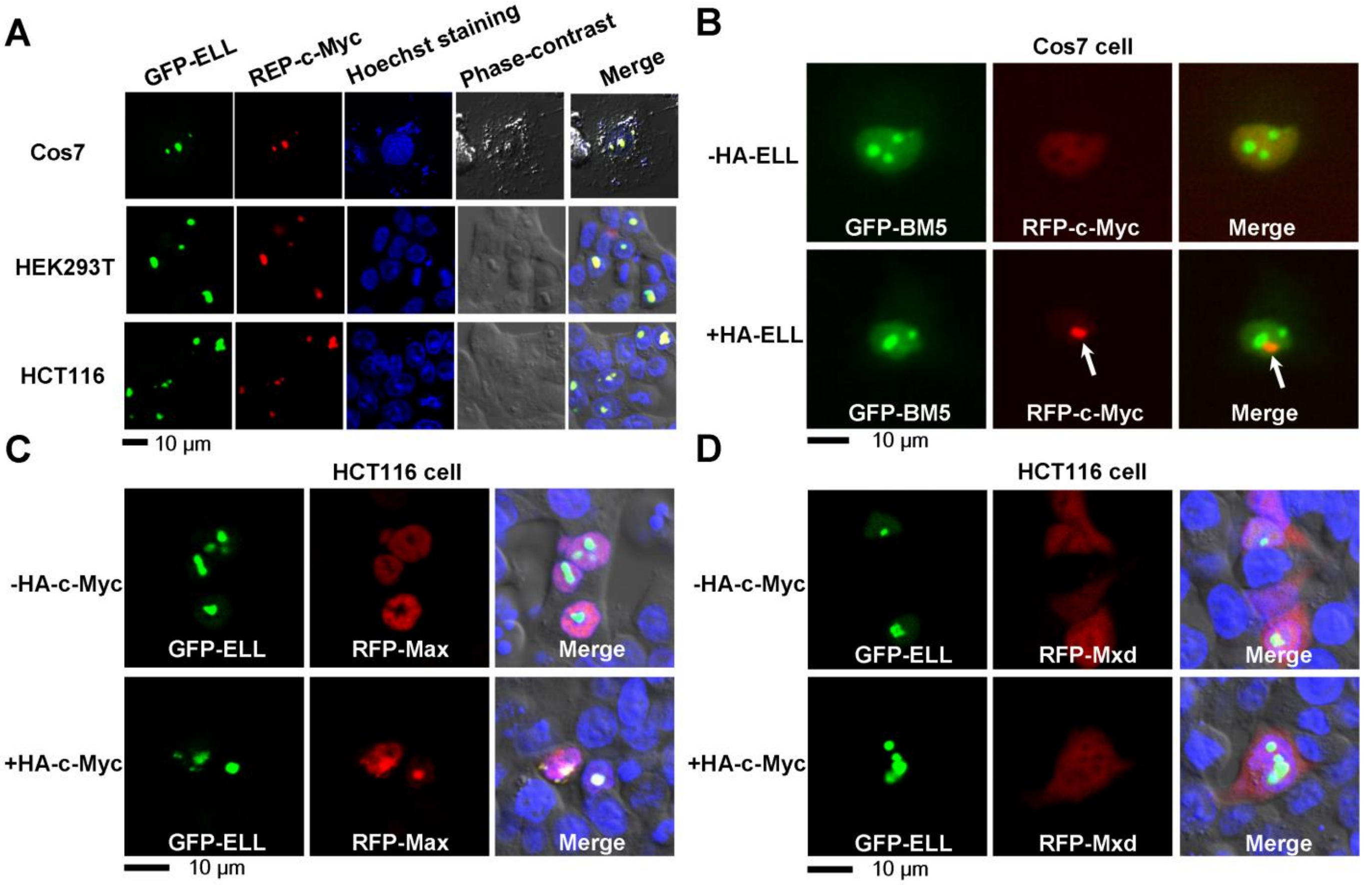

Supplementary Fig. 3. ELL and c-Myc do not co-localize in the nuclei but co-localize in c-Myc/Max complex. (A) GFP-ELL co-localizes with RFP-c-Myc in the nuclei of Cos7, HEK293T and HCT116 cells. (B) In the absence of HA-ELL, RFP-c-Myc does not localize to the nucleolus, which is marked by GFP-tagged BM5 (upper panels) in Cos7 cells. In the presence of HA-ELL, RFP-c-Myc is concentrated to form a speckle in the nucleus of Cos7 cells (white arrow), but not at the nucleolus as marked by GFP-BM5 (lower panels). (C) In the absence of HA-c-Myc, GFP-ELL localizes in the nuclei of HCT116 cells, but not co-localizes with RFP-Max (upper panels). In the presence of HA-c-Myc, GFP-ELL and RFP-Max co-localizes in the nuclei of HCT116 cell (lower panels). (D) In the absence/presence of HA-c-Myc, GFP-ELL does not co-localize with RFG-Mxd.

Supplementary Figure 4

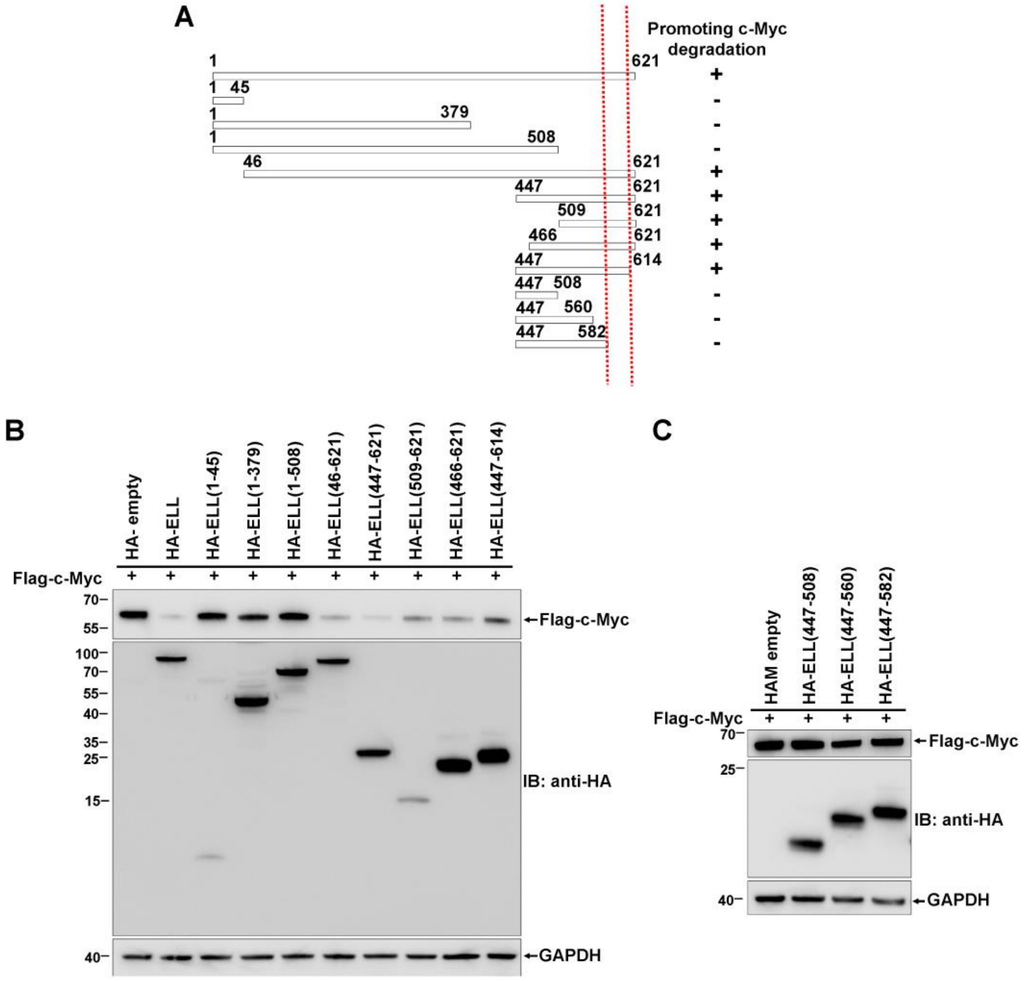

Supplementary Fig. 4. Domain mapping of ELL for promoting c-Myc degradation. (A) Schematic of ELL domains. The truncated domain promoting c-Myc degradation is indicated by plus sign (+); the region (aa 583-614) in ELL required for promoting c-Myc degradation is marked by two red-dash lines. (B, C) The effect of ELL domains on c-Myc degradation is examined by Western blot analysis; Flag-c-Myc is co-transfected with the indicated plasmids in HEK293 cells.

Supplementary Figure 5

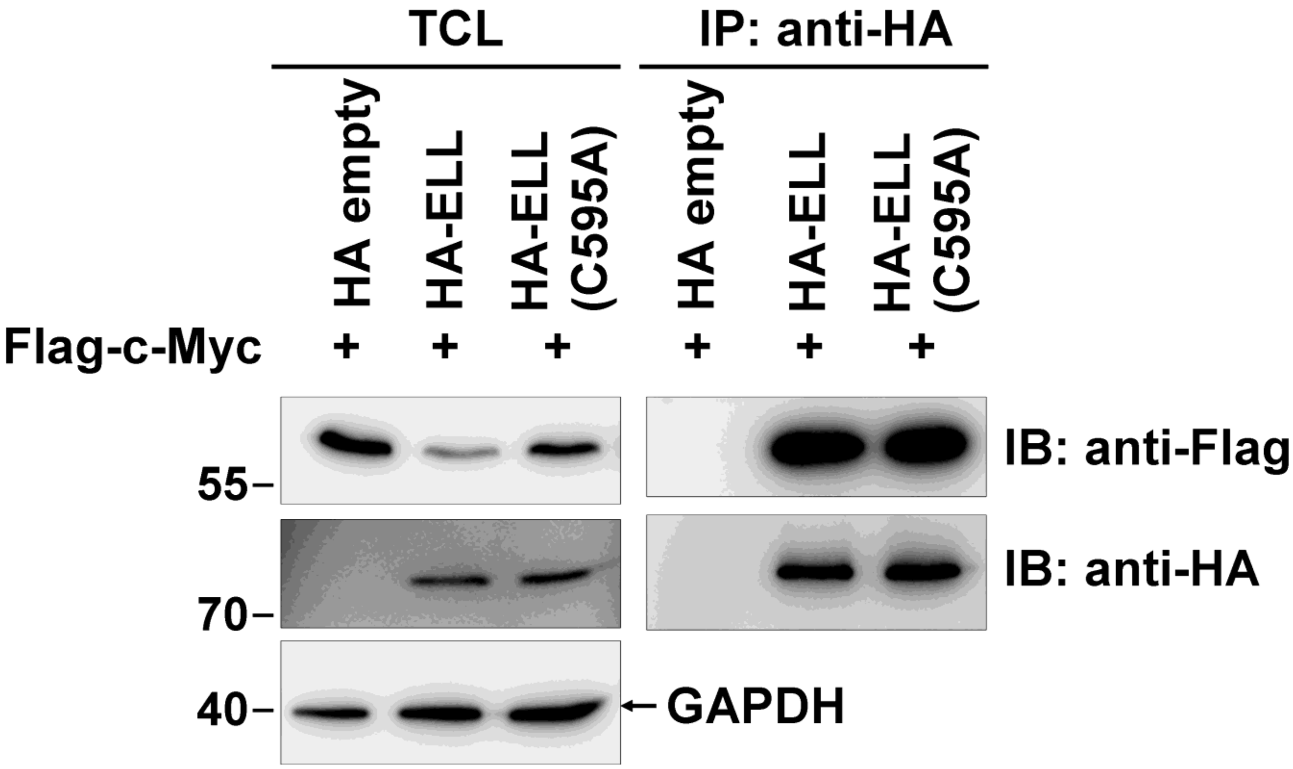

Supplementary Fig. 5. Interaction of c-Myc with ELL mutant (A) Co-immunoprecipitation of human c-Myc with the HA-ELL(C595A) mutant in HEK293T cells transfected with the indicated plasmids.

Supplementary Figure 6

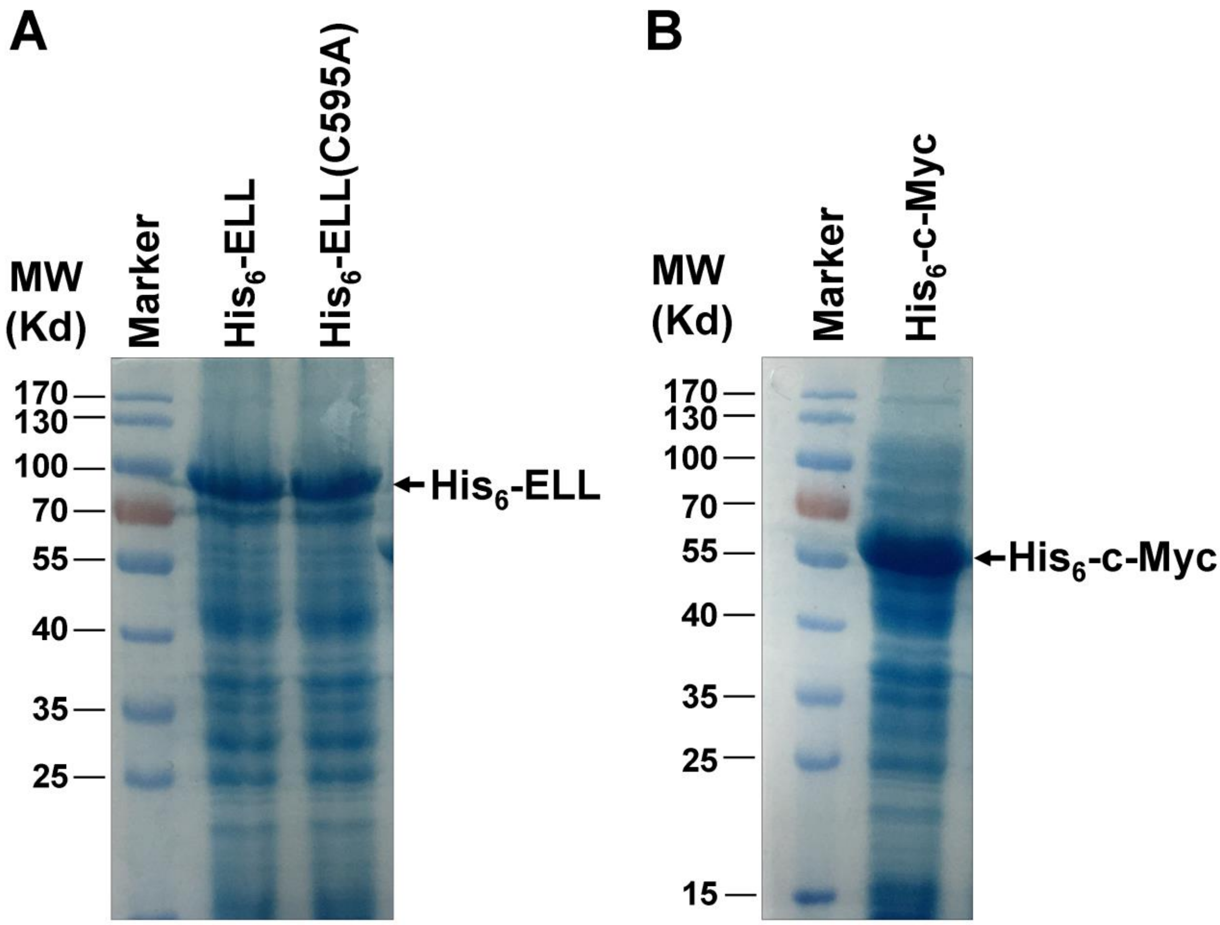

Supplementary Fig. 6. Bacterial expression of His6-ELL, His6-ELL(C595A) and His6-c-Myc..

(A) His6-ELL and His6-ELL(C595A) purified from *E. coli* extracts are examined by SDS-PAGE. (B) His6-c-Myc purified from *E. coli* extracts is examined by SDS-PAGE.

Supplementary Figure 7

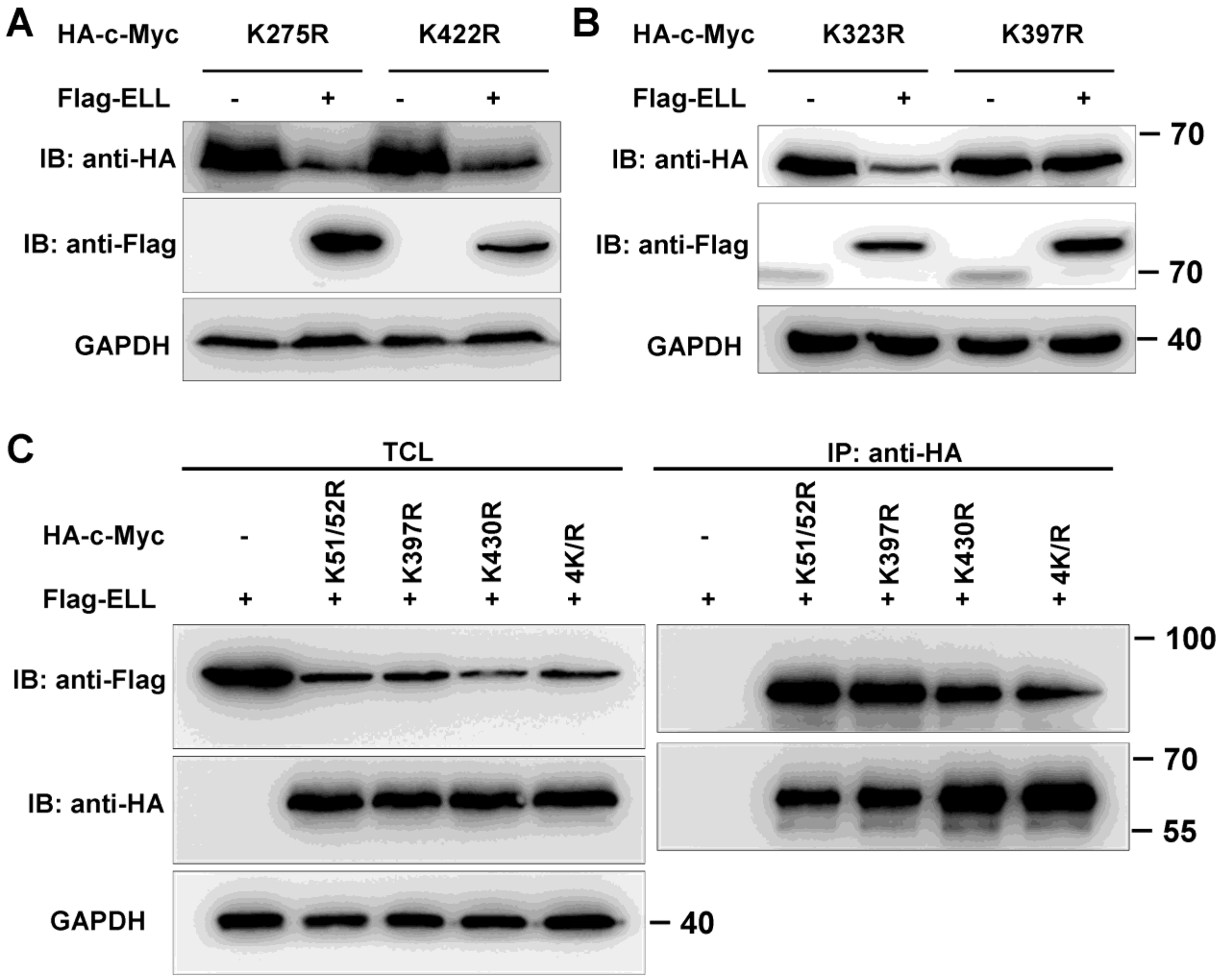

Supplementary Fig. 7. Analysis of ELL-induced degradation and interaction with c-Myc mutants. (A) ELL-induced degradation of K275R and K422R c-Myc mutants is further confirmed. (B) ELL does not induce K397R degradation, but indeed induce K323R degradation. (C) Co-immunoprecipitation of human ELL with the human c-Myc mutants, K51/52R, K397R, K430R and 4K/R, in HEK293T cells transfected with the indicated plasmids.

Supplementary Figure 8

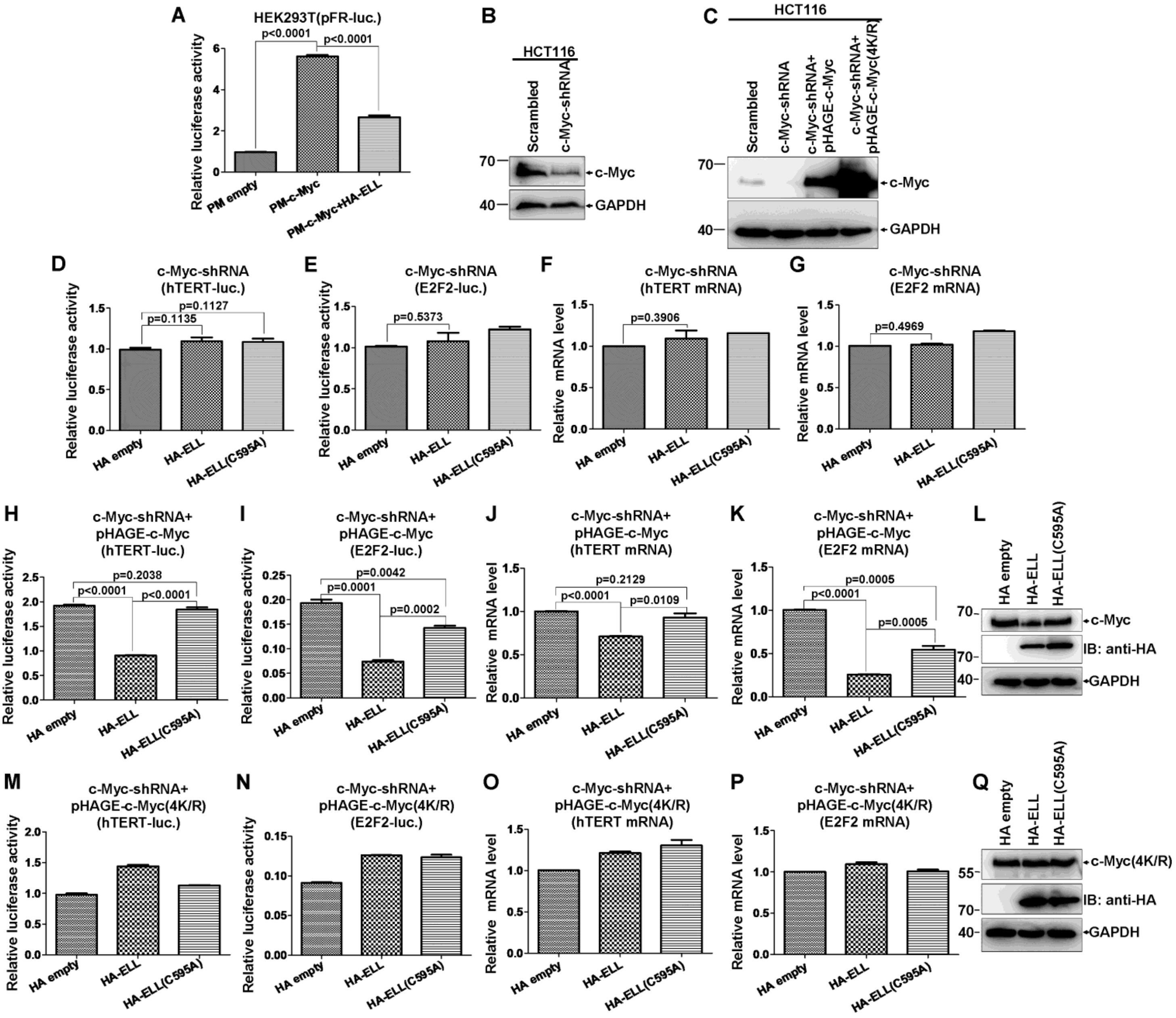

Supplementary Fig. 8. c-Myc is required for ELL-mediated inhibition of downstream targets of *c-Myc*. (A) Overexpression of ELL suppresses c-Myc transcriptional activity. (B) shRNA-mediated knockdown of c-Myc via lentivirus infection in HCT116 cells is confirmed by Western blot analysis. (C) Knockdown of c-Myc and overexpression of wild-type c-Myc or c-Myc(4K/R) mutant is confirmed by Western blot analysis. (D, E) Overexpression of HA-ELL or HA-ELL(C-595A) has no effect on *hTERT* (D) and *E2F2* (E) promoter reporter activity in HCT116 cells after c-Myc knockdown. (F, G) Overexpression of HA-ELL has no effect on *hTERT* (F) and *E2F2* (G) mRNA level in HCT116 cells after c-Myc knockdown. (H, I) The inhibitory effect of ELL on *hTERT* (H) and *E2F2* (I) promoter reporter activity is restored when wild-type c-Myc is re-expressed in HCT116 cells after c-Myc knockdown. (J, K) The inhibitory effect of ELL on *hTERT* (J) and *E2F2* (K) mRNA level is restored when wild-type c-Myc is re-expressed in HCT116 cells after c-Myc knockdown. (L) Overexpression of HA-ELL, HA-ELL(C595A) and c-Myc is confirmed by Western blot analysis. (M, N) The inhibitory effect of ELL on *hTERT* (M) and *E2F2* (N) promoter reporter activity is not restored when the c-Myc(4K/R) mutant is expressed in HCT116 cells after c-Myc knockdown. (O, P) The suppressive effect of ELL on *hTERT* (O) and *E2F2* (P) mRNA level is not restored when c-Myc(4K/R) mutant is expressed in HCT116 cells after c-Myc knockdown. (Q) Overexpression of HA-ELL, HA-ELL(C595A) and c-Myc (4K/R) is confirmed by Western blot analysis. Data are presented as mean $\pm$SEM of three independent experiments performed in triplicate. The statistical analysis was performed using GraphPad Prism 5 (unpaired t-test).

Supplementary Figure 9

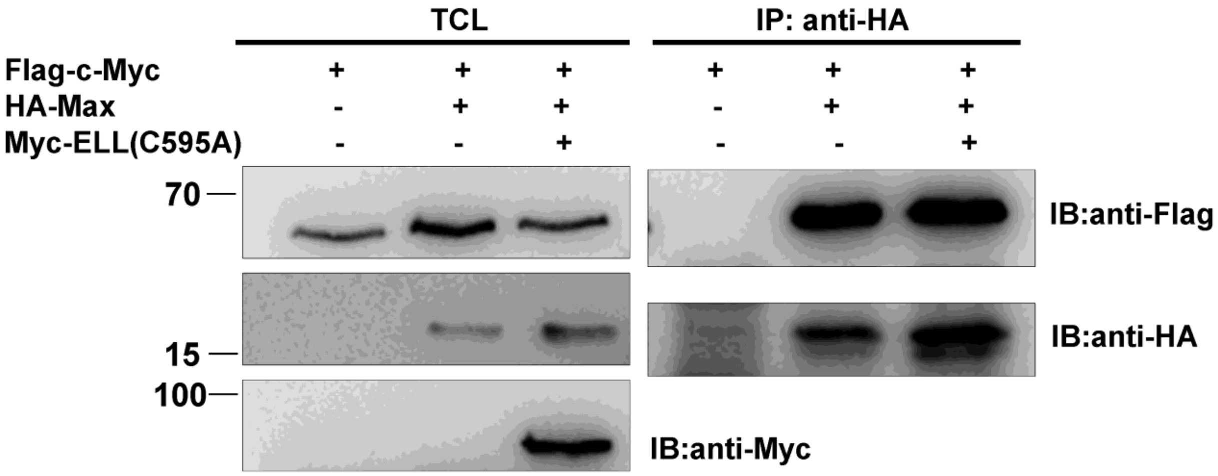

Supplementary Fig. 9. ELL has no obvious effect on the interaction between c-Myc and Max.

Co-immunoprecipitation of c-Myc with Max in HEK293T cells transfected with the indicated plasmids.

Supplementary Figure 10

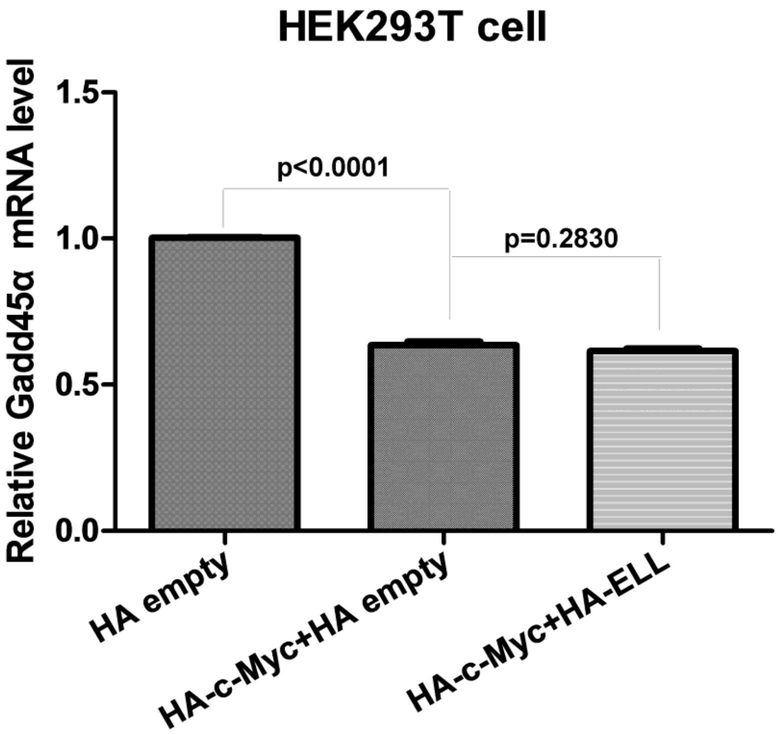

Supplementary Fig. 10. ELL has no effect on c-Myc suppressive function. c-Myc inhibits Gadd45α expression (p<0.0001, *t*-test), but co-transfection of ELL with c-Myc has no obvious effect on c-Myc suppressive function (p=0.2830, *t*-test) in HEK293T cells as revealed by semi-quantitative RT-PCR analysis.

Supplementary Figure 11

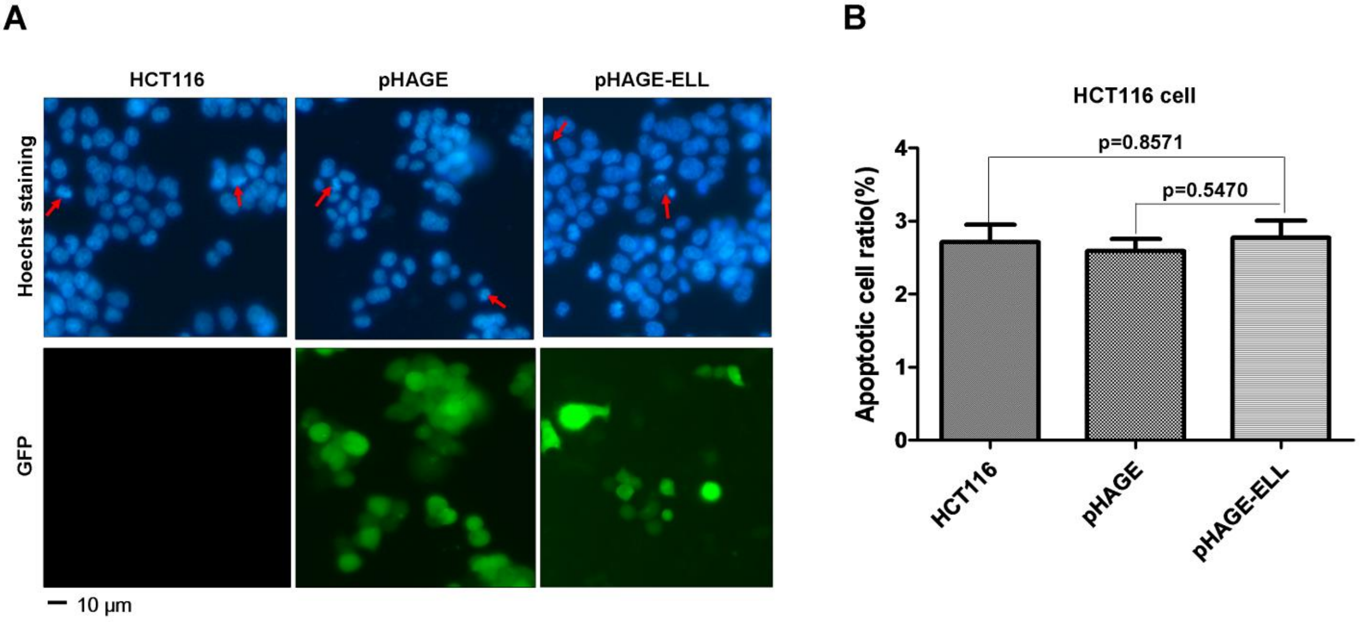

Supplementary Fig. 11. ELL has no effect on cell apoptosis. (A) HCT116 parental cells, HCT116 cells infected with lentivirus control and HCT116 cells infected with ELL-overexpression lentivirus were stained by Hoechst 33342, and the apoptotic cells were countered based on the photographs taken under a fluorescence microscope. (B) quantitative analysis for apoptotic cells. Data are presented as mean $\underline{+}$SEM performed in triplicate. The statistical analysis was performed using GraphPad Prism 5 (unpaired t-test)

Supplementary Figure 12

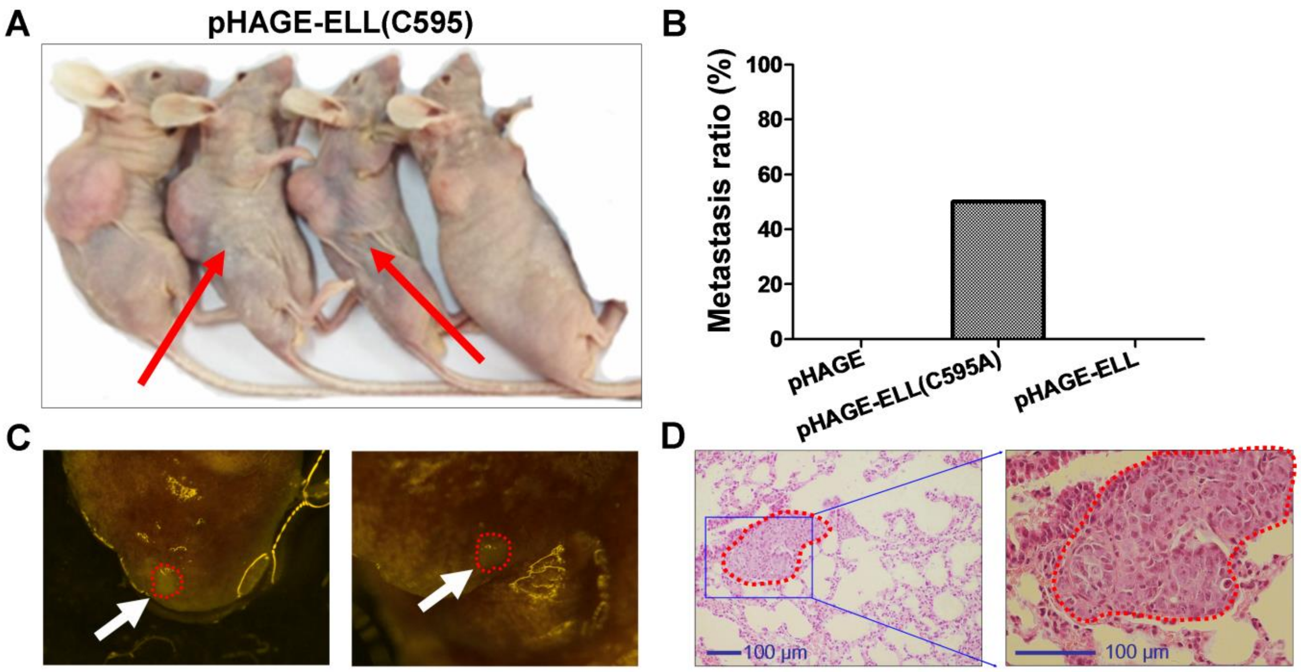

Supplementary Fig. 12. The ELL (C595A) mutant promotes colon cancer metastasis in nude mice. (A) Nude mice with ELL(C595A)-expressing HCT116 xenograft tumors exhibits cachexia (red arrow).(B) quantitative analysis for metastasis ratio. (C) Macro-metastasis (red-dashed circle; white arrow) are observed in the lungs of nude mice (the red arrows marked in A) with ELL(C595A)-expressing tumors. (D) The metastasis in the lungs of nude mice with ELL(C595A)-expressing tumors is confirmed by histological analysis after hematoxyin and eosin staining (red-dashed circle).

Supplementary Figure 13

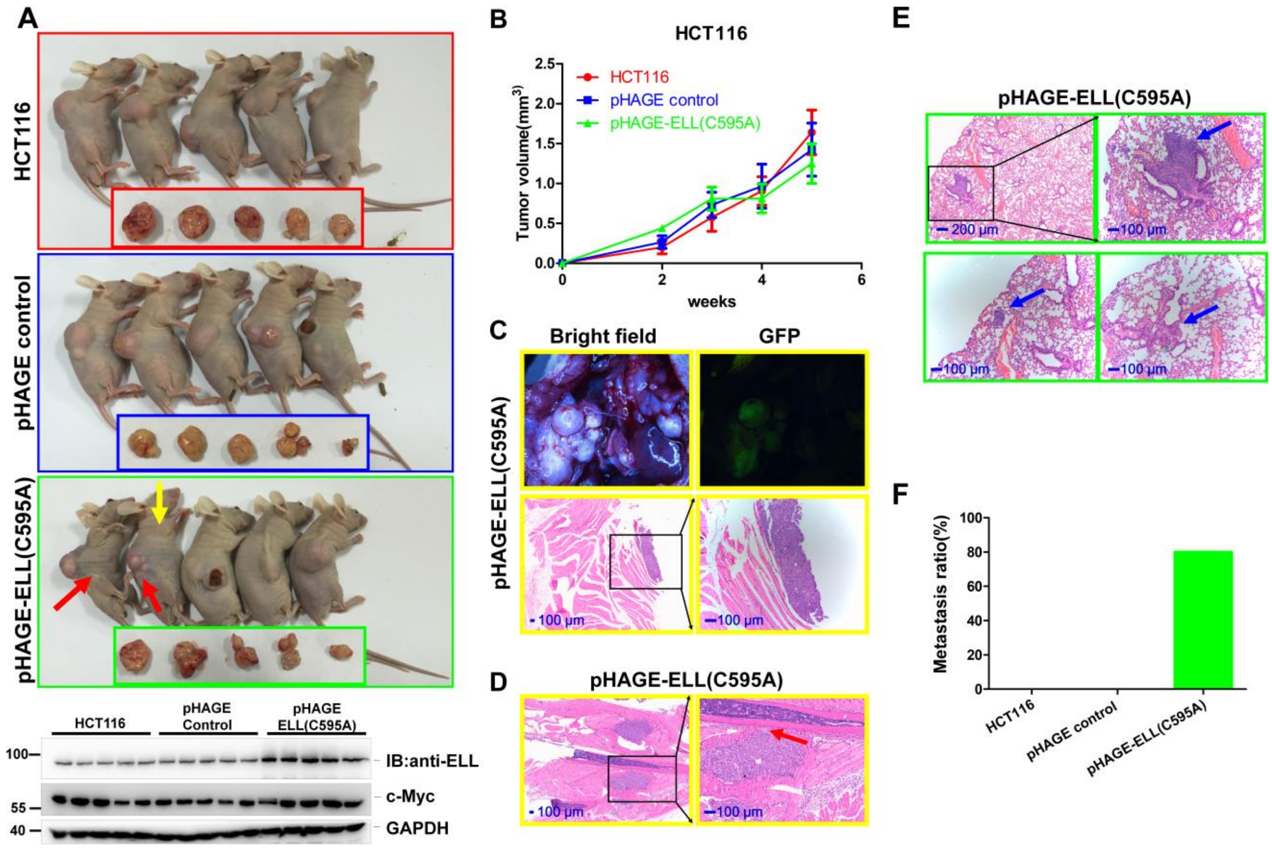

Supplementary Fig. 13. The xenograft tumors derived from HCT116 parental cells, pHAGE control cells and pHAGE-ELL(C595A) cells have similar growth rate, and the ELL (C595A) mutant promotes colon cancer metastasis is further confirmed. (A) The xenograft tumors derived from HCT116 parental cells, pHAGE control cells and pHAGE-ELL(C595A) cells. Two mice with pHAGE-ELL(C595A) tumor exhibit cachexia (red arrows). (**B**) The growth rates of xenograft tumors derived from HCT116 parental cells, pHAGE control cells and pHAGE-ELL(C595A) cells. (**C**) One mice (yellow arrow in A) developed macro-metastasis in the whole chest. The tumor cells are labeled by GFP expression as a result of Lentivirus infection (pHAGE vector contains a GFP marker). (**D**) the mice (yellow arrow in A) with macro-metastasis in the chest exhibits potential bone invasion. (**E**) the micro-metastasis developed in the lung of other three mice with pHAGE-ELL(C595A) is detected by histological analysis (blue arrows). (**F**) quantitative analysis for metastasis ratio.

Supplementary Figure 14

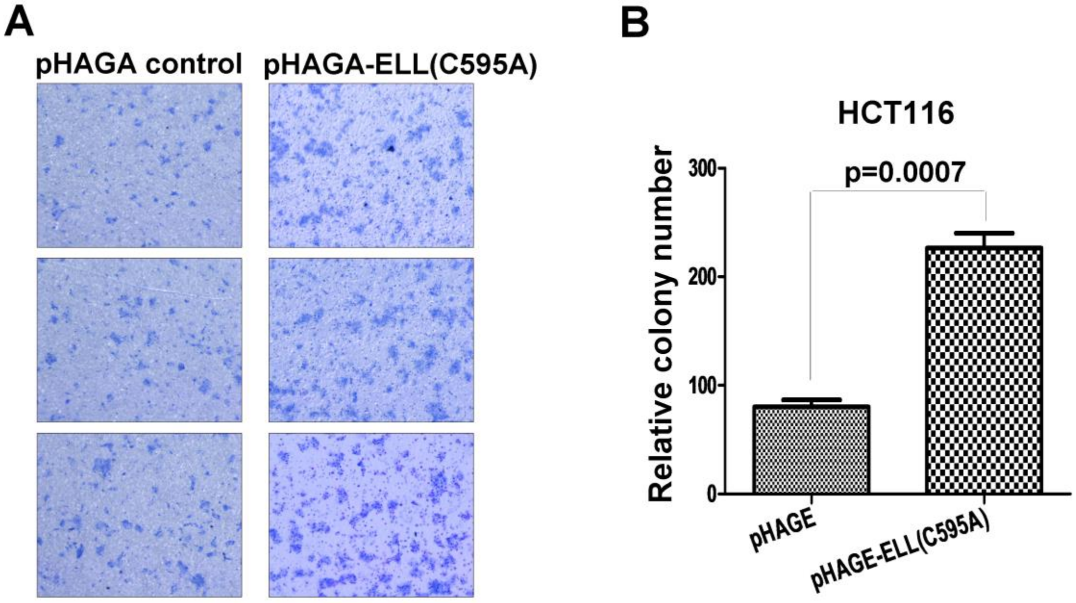

Supplementary Fig. 14. The ELL(C595A) mutant promotes colon cancer cell invasion. (A) HCT116 cells with ELL(C595A) expression exhibited higher invasive capability compared to the control HCT116 cells by the cell invasion assays. (B) Quantitative analysis for the cell invasion assays.

Supplementary Figure 15

A

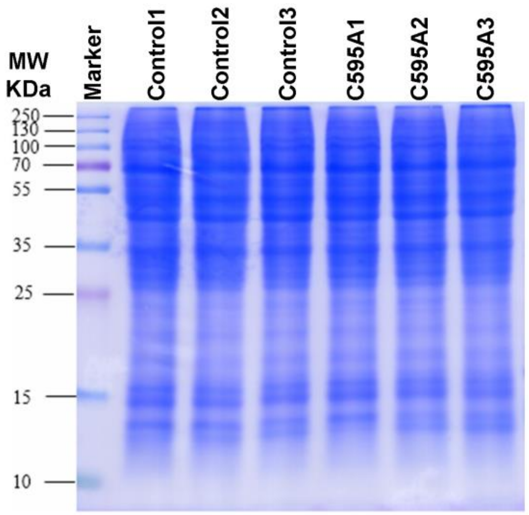

B

The genes up-regulated in the xenograft HCT116 tumors with ELL(C595A) overexpression

Protein ID	Gene	Up-ratio (>1.2)	P-value (<0.05)
Q9UKX2	MYH2	1.299	0.0408497
Q93050	ATP6V0A1	1.221	0.0266267
Q96P70	IPO9	1.38	0.0208679
P17661	DES	1.23	0.0222371
Q9BZH6	WDR11	1.2295	0.0112769
P49006	MARCKSL1	1.308	0.0054785
P50895	BCAM	1.301	0.0200497
P26447	S100A4	1.213	0.0013307
O43688	PPAP2C	1.21	0.0311648
Q8N684	CPSF7	1.2095	0.0048159
O95429	BAG4	1.95	0.0190679

C

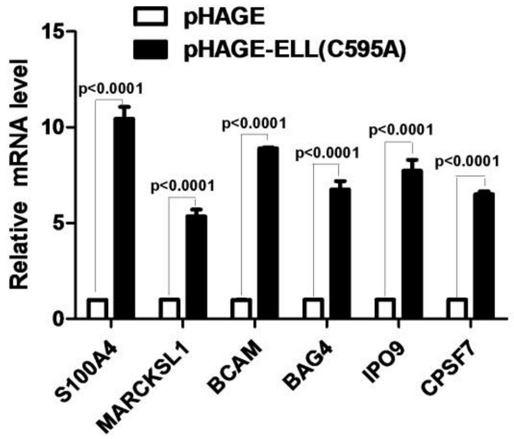

D

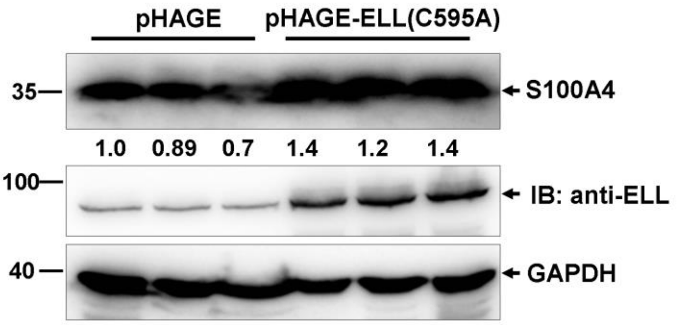

Supplementary Fig. 15. Quantitative analysis of global proteome in xenograft tumors. (A) The total proteins used for quantitative proteomics is examined by SDS-PAGE (12%) (each lane loading 30 μg total protein). (B) The genes up-regulated in the xenograft HCT116 tumors with ELL(C595A) overexpression. (C) The up-regulations of genes in the ELL(C595A) xenograft tumors are confirmed by semi-quantitative RT-PCR analysis. (D) The up-regulation of S100A4 in ELL(C595A) xenograft tumors is confirmed by Western Blot analysis.

Supplementary Figure 16

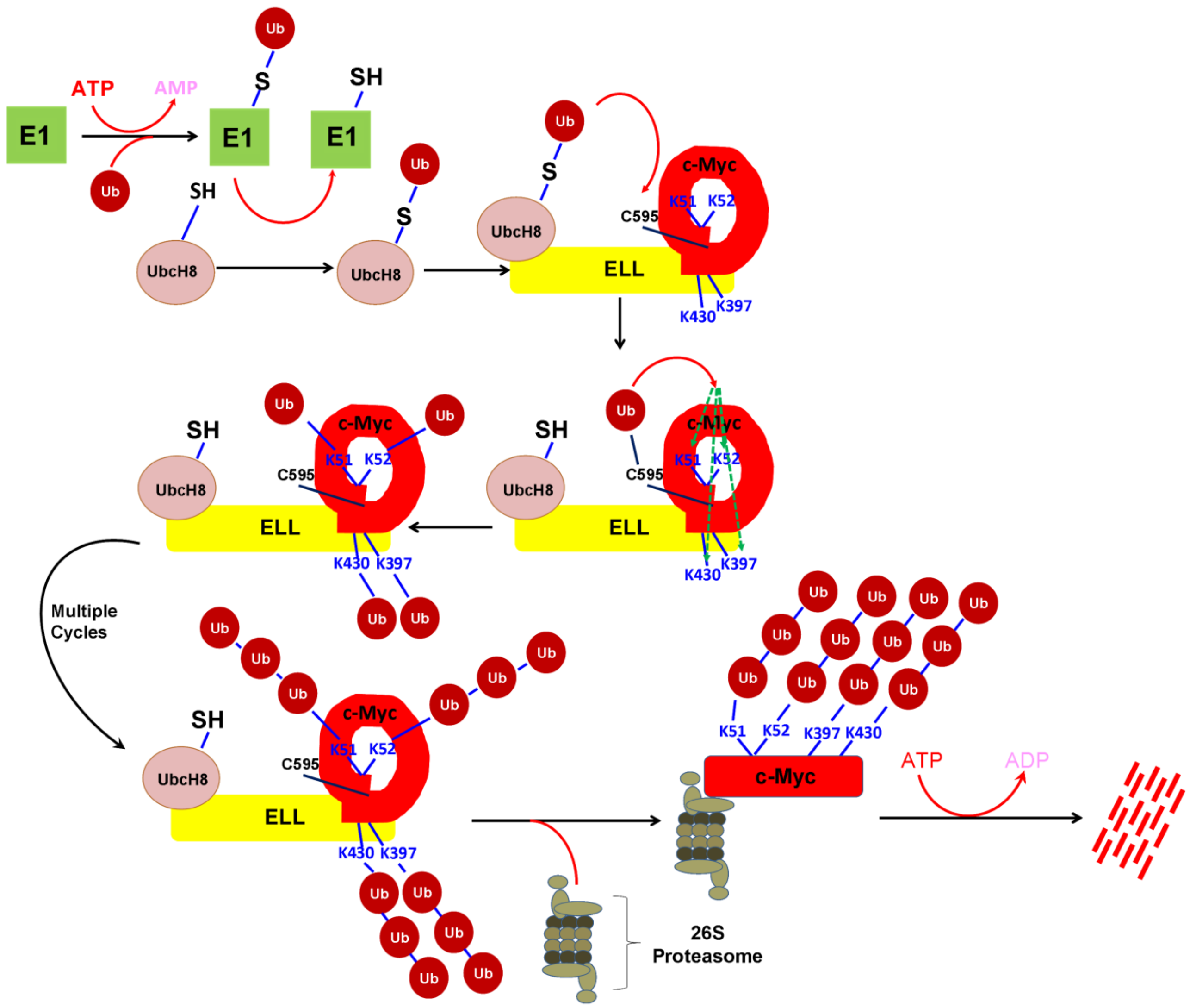

Supplementary Fig. 16. **A model of ELL-mediated c-Myc degradation.** UbcH8 transfers ubiquitin to C595 of ELL, after which ELL transfers ubiquitin from C595 to K51/52, K397 and K430 of c-Myc. After multiple cycles, ELL catalyzes the formation of K48-linked polyubiquitin chains at K51/52, K397 and K430 of c-Myc, which are recognized by the 26S proteasome and result in c-Myc degradation.

Supplementary Figure 17

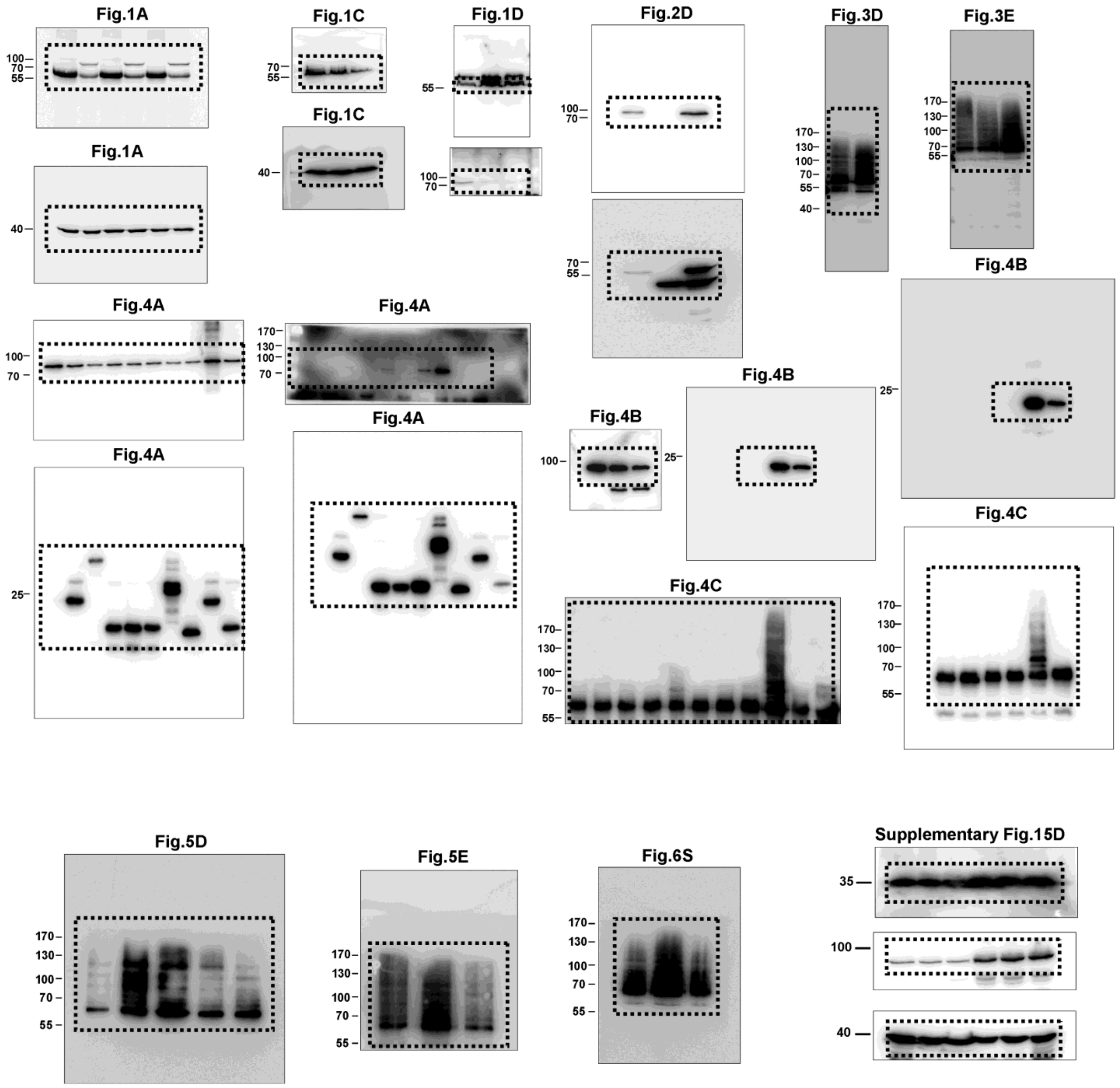

Supplementary Fig.17. The original full immunoblot images utilized in Figure 1, Figure 2, Figure 3, Figure 4, Figure 5, and Supplementary Figure 15

Supplementary Table 1. **The primers used for cloning eleven E2 ubiquitin-conjugating enzyme**

Gene	Sense	Antisense
UbcH1	5'-TCGAATTCCTATGGCCAACATCGC GGTGCAGCGAA-3'	5'-TCCTCGAGTCAGTTACTCAGAA GCAATTCT-3'
UbcH2	5'-TCAGATCTCTATGTCATCTCCCAGT CCG-3'	5'-TACTCGAGCTACAACTCCATATC CT-3'
UbcH3	5'-TCAGATCTCTATGGCTCGGCCGCT AGTG-3'	5'-TAGCGGCCGCTCAGGACTCCTC CGTGCC-3'
UbcH5a	5'-TCAGATCTCTATGGCGCTGAAGAG GATT-3'	5'-TAGCGGCCGCTTACATTGCATAT TTCTG-3'
UbcH5b	5'-TCAGATCTCTATGGCTCTGAAGAG AATCC-3'	5'-TAGCGGCCGCTTACATCGCATA CTTCT-3'
UbcH5c	5'-TCAGATCTCTATGGCGCTGAAACG GATTA-3'	5'-TAGCGGCCGCTCACATGGCATA CTTCTGAGT-3'
UbcH6	5'-TCAGATCTCTATGTCGGATGACGA TTC-3'	5'-TAGCGGCCGCTTATGTAGCGTAT CTCT-3'
UbcH7	5'-TCGAATTCCTATGGCGGCCAGCAG GAGGCTGAT-3'	5'-TAGCGGCCGCTTAGTCCACAGG TCGCTTTTCCCCATAT-3'
UbcH8	5'-TCGAATTCCTATGATGGCGAGCAT GCGA-3'	5'-TACTCGAGTTAGGAGGGCCGGT CCA-3'
UbcH10	5'-TCAGATCTCTATGGCTTCCCAAAA CCGC-3'	5'-TAGCGGCCGCTCAGGGCTCCTG GCTGGT-3'
Ubc13	5'-TAAGATCTATATGGCCGGGCTGCC CCGC-3'	5'-TAGCGGCCGCTTAAATATTATTC ATGGC-3'

Supplementary Table 2. The primers used for confirming gene up-regulation in xenograft HCT116 tumors with ELL(C595A) overexpression by semi-quantitative RT-PCR

Gene	Sense	Antisense
BAG4	5'-TGCACCTGGTTATACTCAGACC-3	5'-TCCATAAGGATAATGGGGCAGGG-3'
BCAM	5'-AGGGCTACATGACCAGCCGCA -3'	5'-AGGGCTACATGACCAGCCGCA-3'
CPSF7	5'-TGAGGTGGTGGTAGCCTCTGAA -3'	5'-TCACTAGAATCTCGGGAATGGG-3'
IPO9	5'-ACTGAGCACTGGTGGAAGATCCA -3'	5'-GACATAGCAACAGTGAACCGAC-3'
MARCKSL1	5'-CCAACGGCCAGGAGAATGGCCA -3'	5'-CTTCTTCTTGGGGGTCTCCT -3'
S100A4	5'-GTGTCCACCTTCCACAAGTACT -3'	5'-TCAGCTTCTGGAAAGCAGCTTC-3'
